# Targeted degradation of ⍺-synuclein aggregates in Parkinson’s disease using the AUTOTAC technology

**DOI:** 10.1186/s13024-023-00630-7

**Published:** 2023-06-24

**Authors:** Jihoon Lee, Ki Woon Sung, Eun-Jin Bae, Dabin Yoon, Dasarang Kim, Jin Saem Lee, Da-ha Park, Daniel Youngjae Park, Su Ran Mun, Soon Chul Kwon, Hye Yeon Kim, Joo-Ok Min, Seung-Jae Lee, Young Ho Suh, Yong Tae Kwon

**Affiliations:** 1 Cellular Degradation Biology Center, College of Medicine, Seoul National University, Seoul, 03080 Republic of Korea; 2 Department of Biomedical Sciences, College of Medicine, Seoul National University, Seoul, 03080 Republic of Korea; 3 AUTOTAC Bio Inc., Changkyunggung-Ro 254, Jongno-Gu, Seoul, 03077 Republic of Korea; 4 Neuroscience Research Institute, College of Medicine, Seoul National University, Seoul, 03080 Republic of Korea; 5 Department of Physical Education, Sejong University, Seoul, 05006 Republic of Korea; 6 Neuramedy Co. Ltd, Seoul, 04796 Republic of Korea; 7 Convergence Research Center for Dementia, Seoul National University Medical Research Center, Seoul, 03080 Republic of Korea; 8 Ischemic/Hypoxic Disease Institute, College of Medicine, Seoul National University, Seoul, 03080 Republic of Korea

**Keywords:** The autophagy-lysosome system, The N-degron pathway, p62/SQSTM1/Sequestosome-1, Macroautophagy, Lysosome, Targeted protein degradation

## Abstract

**Background:**

There are currently no disease-modifying therapeutics for Parkinson’s disease (PD). Although extensive efforts were undertaken to develop therapeutic approaches to delay the symptoms of PD, untreated α-synuclein (α-syn) aggregates cause cellular toxicity and stimulate further disease progression. PROTAC (Proteolysis-Targeting Chimera) has drawn attention as a therapeutic modality to target α-syn. However, no PROTACs have yet shown to selectively degrade α-syn aggregates mainly owing to the limited capacity of the proteasome to degrade aggregates, necessitating the development of novel approaches to fundamentally eliminate α-syn aggregates.

**Methods:**

We employed AUTOTAC (Autophagy-Targeting Chimera), a macroautophagy-based targeted protein degradation (TPD) platform developed in our earlier studies. A series of AUTOTAC chemicals was synthesized as chimeras that bind both α-syn aggregates and p62/SQSTM1/Sequestosome-1, an autophagic receptor. The efficacy of Autotacs was evaluated to target α-syn aggregates to phagophores and subsequently lysosomes for hydrolysis via p62-dependent macroautophagy. The target engagement was monitored by oligomerization and localization of p62 and autophagic markers. The therapeutic efficacy to rescue PD symptoms was characterized in cultured cells and mice. The PK/PD (pharmacokinetics/pharmacodynamics) profiles were investigated to develop an oral drug for PD.

**Results:**

ATC161 induced selective degradation of α-syn aggregates at DC_50_ of ~ 100 nM. No apparent degradation was observed with monomeric α-syn. ATC161 mediated the targeting of α-syn aggregates to p62 by binding the ZZ domain and accelerating p62 self-polymerization. These p62-cargo complexes were delivered to autophagic membranes for lysosomal degradation. In PD cellular models, ATC161 exhibited therapeutic efficacy to reduce cell-to-cell transmission of α-syn and to rescue cells from the damages in DNA and mitochondria. In PD mice established by injecting α-syn preformed fibrils (PFFs) into brain striata via stereotaxic surgery, oral administration of ATC161 at 10 mg/kg induced the degradation of α-syn aggregates and reduced their propagation. ATC161 also mitigated the associated glial inflammatory response and improved muscle strength and locomotive activity.

**Conclusion:**

AUTOTAC provides a platform to develop drugs for PD. ATC161, an oral drug with excellent PK/PD profiles, induces selective degradation of α-syn aggregates in vitro and in vivo. We suggest that ATC161 is a disease-modifying drug that degrades the pathogenic cause of PD.

**Supplementary Information:**

The online version contains supplementary material available at 10.1186/s13024-023-00630-7.

## Background

Parkinson’s disease (PD) is one of the most prevalent neurodegenerative disorders, affecting 2–3% of the population above 65 years of age [1, 2]. One prominent hallmark of PD is the neuro-aggregation of misfolded α-synuclein (α-syn), forming Lewy bodies (LBs) in substantia nigra, leading to subsequent dopaminergic neuronal degeneration [3]. Autosomal dominant forms of PD are caused due to point mutations (A30P, E46K, H50Q, G51D, A53T, and A53E) and duplication or triplication of the *SNCA* gene, some of which are known to directly enhance the propensity for the aggregation of α-syn [4–6]. Moreover, converging evidence from studies with animal PD models have indicated that exposure to pesticides such as rotenone and paraquat promotes the formation of α-syn inclusions associated with PD [7–9]. These α-syn aggregates and the formation of LBs are known to exert cellular stress [10, 11]. Given the evidence that α-syn aggregates are central to the etiology of familial and sporadic PD, an ultimate therapeutic strategy to treat PD would be targeted degradation of α-syn aggregates from neurons. Nevertheless, current pharmacological treatments rely on dopaminergic medications such as levodopa, dopamine agonists, and MAO-B inhibitors to achieve the transient relief of motor symptoms characterized by tremor, bradykinesia, and rigidity [12, 13]. Levodopa, the most widely used dopaminergic medication, is known to be accompanied with motor complications when taken in a long term [14]. Moreover, no current clinical interventions are designated to be beneficial for delaying or preventing PD disease progression [15], necessitating novel therapeutic approaches that can fundamentally alter the disease course of PD.

Recent studies showed that the degradation of misfolded α-syn and its aggregates involves the cooperative efforts by the ubiquitin–proteasome system (UPS), chaperone-mediated autophagy (CMA), and macroautophagy (henceforth autophagy) [16]. As the first line of defense for proteostasis, the UPS is responsible for the removal of ubiquitinated or Ser129-phosphorylated α-syn [16, 17]. Although small, soluble oligomers were found to be partially degraded through the UPS, larger oligomeric forms are resistant to the UPS and requires the activity of autophagy for their clearance [18, 19]. The degradative capacity of CMA has been shown with wild-type α-syn but not aggregation-prone α-syn mutants including A30P and A53T as well as wild-type α-syn oligomers [20, 21]. Exacerbating these, α-syn accumulation causes the malfunctioning of degradation pathways and shifts the burden towards autophagy to remove oligomeric or larger aggregates [16]. Moreover, while α-syn inclusions are targeted by autophagy and destined for lysosomal degradation, the basal activity of autophagy in the course of PD pathogenesis was shown to be insufficient to suppress α-syn accumulation [22]. As a consequence, LBs are composed of ubiquitin, E3 ubiquitin-ligases including parkin, autophagic receptors, and chaperones, indicating the battlefield of degradation systems struggling to suppress α-syn accumulation [23–26]. These suggest that therapeutic approaches enhancing such endogenous degradation pathways can restore neuronal homeostasis.

Targeted protein degradation (TPD) has emerged as a therapeutic modality to selectively target pathogenic proteins and their aggregates directly to degradation pathways [27]. The study by Fan et al. reported the development of protein-specific knockdown peptide containing a CMA-targeting motif [28]. This CMA-based knockdown technology showed the targeted degradation of wild-type as well as A53T mutant α-syn in primary cultured neurons at 25 μM and 50 μM, respectively, overcoming the previously known inhibitory effect of mutant α-syn on the lysosomal receptor involved in CMA [20]. Alternatively, the study by Qu et al. utilized the UPS by designing an α-syn-binding fusion peptide containing a proteasome-targeting motif [29]. This synthesized peptide exhibited degradation efficacy in vitro to monomeric α-syn at 10 μM. Amongst proteasomal TPD technologies, PROTAC (proteolysis-targeting chimera) is a widely studied therapeutic approach. The patent by Kargbo et al. reported a set of PROTACs for the degradation of A53T α-syn in HEK293 cells at 1 μM [30]. These studies demonstrate that the emerging TPD technologies enable the degradation of wild-type or mutant α-syn in its monomeric level, implying their therapeutic potential in preventing α-syn aggregation in early stages. However, given the inabilities of the chaperones and the proteasome to unfold α-syn oligomers for degradation and the inner diameter of the proteasome acting as a physical barrier [18, 31, 32], there are still significant questions on how these TPD strategies would tackle oligomeric, fibrillar, or larger aggregates that are sized beyond the capacities of the aforementioned degradation systems. Hence, autophagy-dependent TPD technologies are a valuable tool for the removal of large α-syn aggregates, since the aggregates have been untouchable by the other TPD technologies.

The N-degron pathway is a degradative system, in which the degradation of various cellular materials is governed by the single N-terminal amino acids of proteins, such as arginine (Arg) [33]. This degradation determinant, called the N-degron, is selectively recognized by cognate N-recognins including the E3 ligases UBR1, UBR2, UBR4, and UBR5 which induce substrate ubiquitination leading to proteasomal degradation [34–36]. We have recently identified the autophagic N-degron pathway, in which the autophagic receptor p62/SQSTM1/Sequestosome-1 acts as an N-recognin of Arg/N-degron via its ZZ-domain [37, 38]. When autophagic cargoes such as protein aggregates accumulate in the cell, a set of molecular chaperones are relocated from the endoplasmic reticulum (ER) to the cytosol and Nt-arginylated by ATE1 R-transferases. The resulting Arg/N-degron binds the ZZ domain of p62, which exposes its PB1 and LC3-binding domains, accelerating p62 self-polymerization in complex with protein aggregates and p62 interaction with LC3 on phagophores, leading to lysosomal degradation [37–39]. The autophagic N-degron pathway modulates the lysosomal degradation of various biological materials ranging from protein aggregates and subcellular organelles such as the ER and peroxisome to invading pathogens such as a broad range of bacteria [40–42].

We have recently developed small molecule ligands to the ZZ domain of p62 as a means to induce autophagy by mimicking the biological action of Arg/N-degrons [38, 43], which could potentially be utilized as a basis for therapeutic platform. These p62 agonists were shown to accelerate the autophagic degradation of the ER, ER-residing protein aggregates, the peroxisome, and intracellular bacteria such as multi-drug resistance (MDR) *Mycobacterium tuberculosis* [40–42]. These p62 agonists were used to develop an autophagy-based TPD technology, termed AUTOTAC (autophagy-targeting chimera) that enables targeted proteolysis via p62-dependent autophagy [44]. An Autotac is a small bifunctional molecule composed of a target-binding ligand (TBL) linked to an autophagy targeting ligand (ATL). As a proof-of-concept study, Autotacs were designed, synthesized, and demonstrated to induce the lysosomal degradation of a set of oncoproteins such as androgen receptor, estrogen receptor β, MetAP2, and BRD4 as well as tau aggregates in Alzheimer’s disease (AD) [44].

Here, we designed and synthesized Autotacs that target α-syn aggregates. The selected PD-Autotac compound, ATC161, induces targeted autophagic-lysosomal degradation of α-syn aggregates in a p62-dependent manner, consequently alleviating DNA damage and mitochondrial dysfunction in synucleinopathy. Extending these, we show that ATC161 reduces the propagation of α-syn aggregates and the associated glial immune response within brain regions of mice injected with α-syn preformed fibrils (PFFs). Furthermore, the oral administration of ATC161 prevents the progression of motor deficits in the PD mice. In sum, this study suggests that the targeted degradation of α-syn aggregates is a disease-modifying strategy to treat PD, emphasizing the potential of Autotacs as first-in-class therapeutic agents for proteinopathies.

## Results

### The chemical agonists to p62 rescue PD model cells from autophagic suppression by α-syn aggregates

**Fig. 1:**
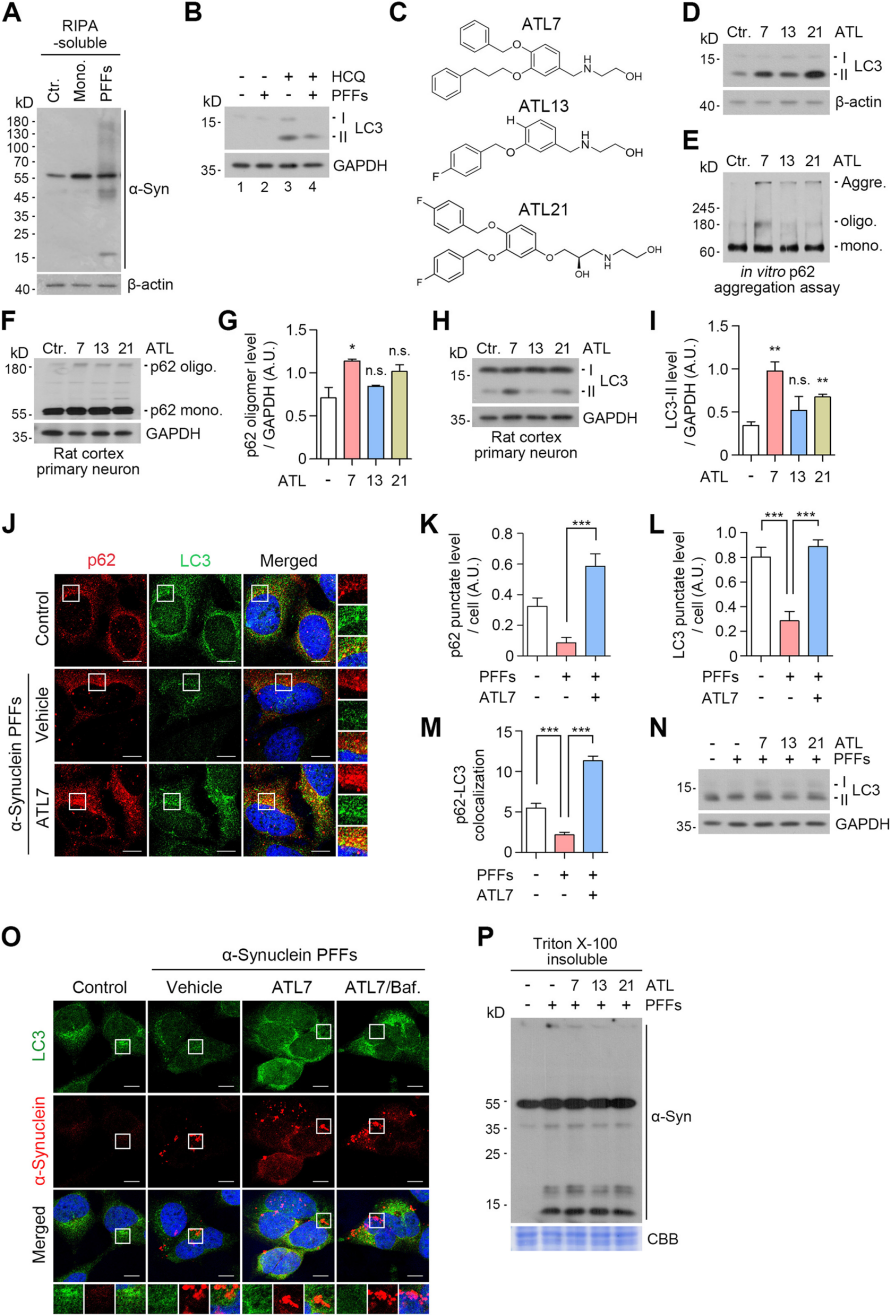
p62 agonists induce autophagy in PD cellular models. **A** Western blot in HEK293A cells transduced with either h-α-syn monomers or h-α-syn PFFs (48 h). **B** Autophagic flux assay in HEK293A cells transduced with h-α-syn PFFs (48 h) upon co-treatment with HCQ (25 μM, 48 h). **C** Structures of ATL compounds. **D** Western blot in HeLa cells, subsequent to ATL treatments (1 μM, 24 h). **E** In vitro p62 aggregation assay in 293 cell lysates. ATLs were treated at final concentration of 1 mM. **F** and **H** Western blots in rat cortex primary neurons. ATLs were treated at 1 μM for 24 h. **G** and **I** Quantifications of F and H (*n* = 3, each). Data are presented as the mean (SEM) where relevant. *P*-values (from two-sided unpaired *t* tests): n.s. (not significant) *P*-value > 0.05, ^*^ *P* ≤ 0.05, ^**^ *P* < 0.01. **J** Immunocytochemistry in HEK293A cells transduced with h-α-syn PFFs (48 h). The cells were then treated with ATL7 (1 μM, 24 h). **K-M** Quantifications of G (*n* = 6–7 for K and L; for M, the numbers of p62^+^LC3^+^ puncta were counted per cell in 20 cells per group). Data are presented as the mean (SEM) where relevant. *P*-values (from two-sided unpaired *t* tests): ^***^ *P* < 0.001. **N** Western blot in HEK293A cells transduced with h-α-syn PFFs (48 h) were treated with ATLs at 1 μM for 24 h. **O** Immunocytochemistry in HEK293A cells transduced with h-α-syn PFFs (48 h), subsequently treated with ATL7 (1 μM, 24 h) and baf. A1 (200 nM, 6 h). **P** Western blot of Triton X-100 insoluble fraction in HEK293A cells. The cells were transduced with h-α-syn PFFs (48 h) followed by treatment with ATLs at 1 μM for 24 h. All the scale bars in this figure represent 10 μm

During the pathogenesis of neurodegeneration, the activities of proteolytic pathways such as the UPS and macroautophagy can be impaired by pathogenic protein aggregates, forming an aggravating cycle between proteolytic machinery and aggregates [45]. To characterize the functional relationship between α-syn aggregates and autophagy, we established a PD cellular model that produces excessive α-syn aggregates by transducing preformed fibrils (PFFs) of human α-syn into HEK293A cells. Immunoblotting analyses confirmed that the cells transduced with α-syn PFFs accumulated SDS-resistant α-syn aggregates in addition to its monomeric species, by accumulating endogenous α-syn (Fig. 1A and S2A). Concurrently, the autophagy flux assay showed that the α-syn aggregation correlated to decreased synthesis of LC3-I and lipidation into LC3-II when lysosomal degradation was blocked by hydroxychloroquine (HCQ) (Fig. 1B, lane 3 vs 4). When visualized using immunocytochemistry, α-syn aggregates caused a marked reduction in the level of LC3^+^ autophagic membranes (Fig. 1J and L). These results confirmed that the aggregation of α-syn impairs the formation of autophagic membranes, supporting the previous findings that α-syn accumulation suppresses autophagic activities [46, 47].

Despite extensive efforts to degrade α-syn aggregates by boosting various autophagic pathways [48, 49], there are currently no therapeutic means to selectively eradicate α-syn aggregates in PD. We therefore asked whether chemical activation of p62-dependent selective autophagy induces the degradation of α-syn aggregates. Our previous work has developed small molecule agonists to the ZZ domain of p62 and demonstrated their activities to induce self-polymerization of p62 and to accelerate autophagic flux of cellular proteins [38, 43]. To develop p62 agonists with improved efficacies in autophagy activation and pharmacological properties, the prototype compounds were modified through SAR (structure–activity relationship) approaches. Following initial screening based on p62 activation and α-syn degradation (data not shown), three compounds were used in this study (Fig. 1C). In addition to the shared efficacy to activate p62 in macroautophagy, ATL7 with a size of 391.5 Da exhibited a B/P ratio of ~ 3.0 (AUC_last_ 722.9 h*ng/g in brain vs 173.4 in plasma) (Fig. S1A). ATL13 (275.3 Da) exhibited pharmacological properties suitable for drug development (Fig. S1A) and, therefore, served as an ATL in Autotac compounds. ATL21 (459.5 Da) exhibited a high oral bioavailability of AUC_last_ 2586 h*ng/ml (Fig. S1A). These compounds accelerated the lipidation of LC3-I into LC3-II in HeLa cells at approximately 1 μM (Fig. 1D).

We assessed the relative activities of ATL7, ATL13, and ATL21 to induce the self-oligomerization of p62 and the synthesis and lipidation of LC3 in rat cortex primary neurons. The highest activities were observed with ATL7 (Fig. 1E-I). Consistently, ATL7 stimulated the formation of p62^+^ as well as LC3^+^ cytosolic puncta to the levels significantly higher than those in normally growing HEK293A cells (Fig. 1J-L). The number of p62^+^LC3^+^ colocalizing puncta was increased by approximately 81% (Fig. 1M), suggesting that ATL7 boosted up the activity of p62-dependent macroautophagy in HEK293A cells challenged with excessive α-syn aggregates. To further validate these results, human α-syn was overexpressed from adenovirus in monkey kidney-derived fibroblast-like COS7 cells, and their aggregation was induced by treating rotenone, a mitochondrial complex I inhibitor. Immunoblotting analyses showed that ATL7 more efficiently promoted the lipidation of LC3 than the other compounds (Fig. S2B, lanes 3 ~ 6). A similar result was also observed in PFF-transduced cells (Fig. 1N). These results suggest that ATL7 restores and boosts up p62-dependent macroautophagy in PD cellular models to the levels even higher than those in normal cells.

Next, we determined whether ATL7 can induce the degradation of α-syn aggregates in PD cellular models. When the colocalization of α-syn with LC3 was monitored using immunocytochemistry analyses, ATL7 did not show significant activities to target α-syn to LC3^+^ autophagic membranes both in the absence and presence of bafilomycin A1, an inhibitor of vacuolar H^+^-ATPase in the lysosome (Fig. 1O). Similar results were observed in each the rotenone-induced and PFF-transduced PD models, in which insoluble proteins separated by using Triton X-100 showed no significant degradation of α-syn aggregates when cells were treated with the p62 agonists (Fig. 1P and S2C). These results suggest that the chemical activation of p62 alone may not be sufficient to degrade α-syn aggregates.

### Design and synthesis of Autotac compounds for α-syn aggregates

We have recently developed the AUTOTAC platform that enables the development of therapeutics for targeted protein degradation via p62-dependent autophagy [44]. An Autotac compound employs a chimeric molecule composed of a target-binding ligand (TBL) linked to an autophagy-targeting ligand (ATL) that binds the ZZ domain of p62. To selectively target α-syn aggregates for lysosomal degradation, we designed and synthesized a set of Autotac compounds based on different rationales when choosing TBLs (Fig. S1B). *First*, ATC161 employed anle138b that binds pre-aggregates of α-syn with K_d_ of approximately 190 nM by recognizing their oligomeric signatures and, thus, inhibits the aggregation of α-syn [44, 50]. Anle138b is currently under Phase 1b clinical trial with PD patients (NCT04685265). *Second*, ATC162 employed baicalein, a flavonoid compound that binds to Tyr residues of α-syn with K_d_ of 500 nM [51]. Based on the activity to inhibit α-syn fibrillization and aggregation and application in neurological diseases, baicalein entered two Phase 1 trials [52]. *Third*, ATC163 employed resveratrol, a natural phenol that binds to α-syn amyloids with K_d_ of 36.8 μM by potential interactions with β-sheet rich structures [53]. Resveratrol was shown to inhibit α-syn aggregation in vitro and to improve motor performance in PD-modeled mice [54]. *Fourth*, ATC164 employed PBA (4-phenylbutyric acid), an FDA-approved drug for children with urea cycle disorders, which also exhibited anti-aggregating properties to α-syn [55, 56]. We selected these TBLs as a strategy to study how their binding characteristics or aggregation-inhibiting profiles to α-syn affect autophagy-inducing and degradative efficacies in the concert with ATLs.

### The AUTOTAC technology enables the targeted degradation of α-syn aggregates

**Fig. 2:**
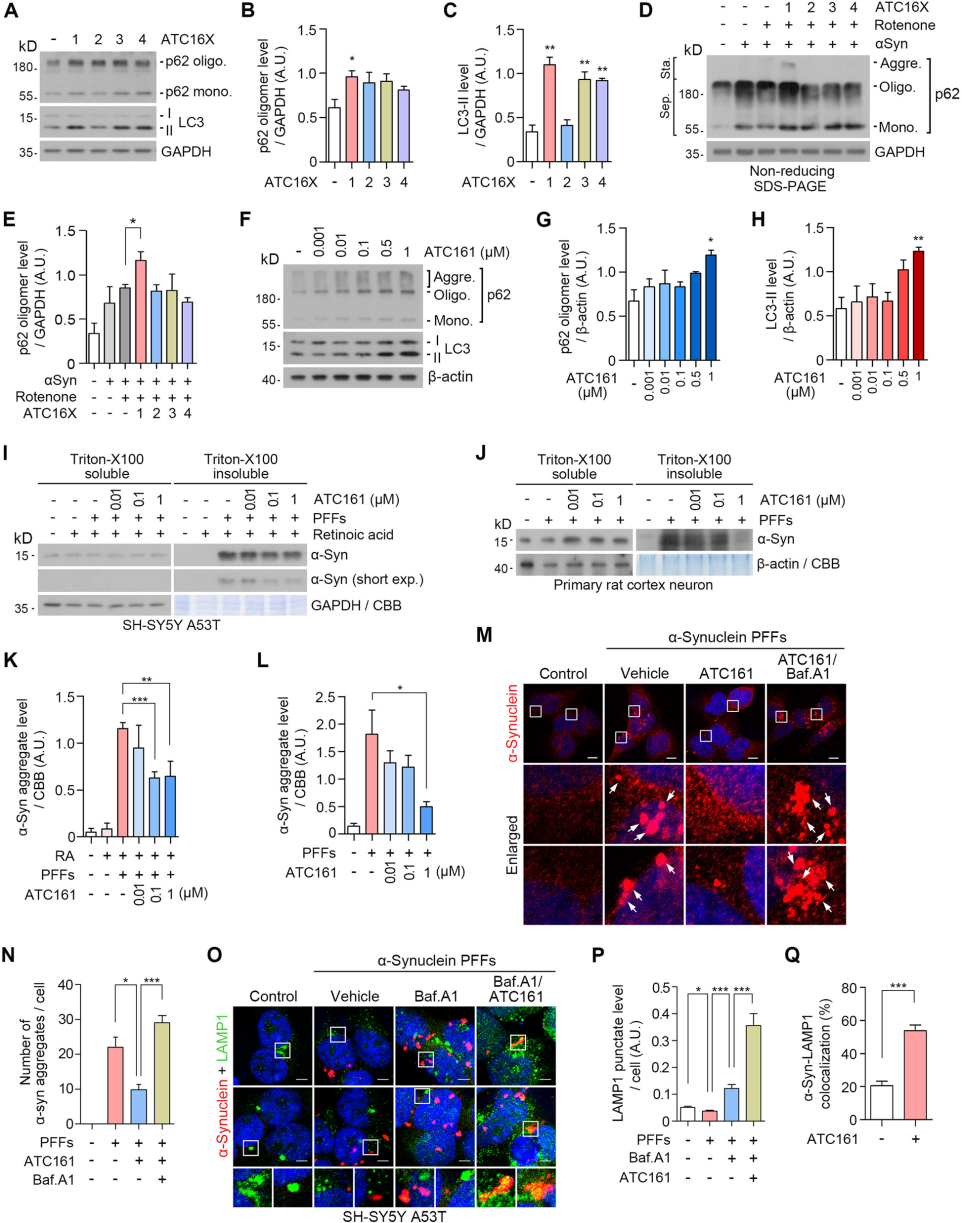
The AUTOTAC technology enables the development of target degraders for α-syn aggregates. **A** Western blot in HeLa cells treated with Autotacs (1 μM, 24 h). **B** and **C** quantifications of A (*n* = 3). Data are presented as the mean (SEM) where relevant. *P*-values (from two-sided unpaired *t* tests): ^*^ *P* ≤ 0.05, ^**^ *P* < 0.01. **D** Non-reducing SDS-PAGE in the previously modelled COS7 cells. **E** Quantification of D (*n* = 3). Data are presented as the mean (SEM) where relevant. *P*-value (from a two-sided unpaired *t* test): ^*^ *P* ≤ 0.05. **F** Western blot in HeLa cells treated with ATC161 in a concentration-dependent manner. **G** and **H** Quantifications of F (*n* = 3). Data are presented as the mean (SEM) where relevant. *P*-values (from two-sided unpaired *t* tests): ^*^ *P* ≤ 0.05, ^**^ *P* < 0.01. **I** Triton-X100 fractionation assay in SH-SY5Y α-syn A53T cells. **J** Identical as I but in primary rat cortex neurons. **K** and **L**, quantifications of I and J (*n* = 3, each), respectively. Data are presented as the mean (SEM) where relevant. *P*-values (from two-sided unpaired *t* tests): ^*^ *P* ≤ 0.05, ^**^ *P* < 0.01, ^***^ *P* < 0.001. **M** Immunocytochemistry in HEK293A cells transduced with h-α-syn PFFs, followed by treatment with ATC161 (1 μM, 24 h) and co-treatment with Baf. A1 (200 nM, 24 h). Scale bars represent 10 μm. **N** Quantification of M (*n* = 3–4). Numbers of α-syn^+^ inclusions were counted per cell. Data are presented as the mean (SEM) where relevant. *P*-values (from two-sided unpaired *t* tests): ^*^ *P* ≤ 0.05, ^***^ *P* < 0.001. **O** Immunocytochemistry in SH-SY5Y α-syn A53T cells transduced with h-α-syn PFFs (48 h), followed by treatment with Baf. A1 (200 nM, 6 h) and co-treatment with ATC161 (1 μM, 6 h). Scale bars represent 5 μm. **P** and **Q** Quantifications of O (*n* = 6–7 and *n* = 7, respectively). For Q, the percentages of α-syn^+^ inclusions targeted to LAMP1^+^ puncta were calculated. Data are presented as the mean (SEM) where relevant. *P*-values (from two-sided unpaired *t* tests): ^*^ *P* ≤ 0.05, ^***^ *P* < 0.001

To test whether the Autotac compounds induce the conformational change of p62, we monitored the level of p62 oligomerization. HeLa cells treated with the compounds at 1 μM were subjected to immunoblotting analyses. When oligomers and high-molecular weight species were separated from monomers, ATC161 (anle138b) most efficiently facilitated p62 oligomerization compared with the other Autotac compounds (Fig. 2A and B). As an alternative assay, we employed the COS7 cellular PD model. Non-reducing SDS-PAGE showed that only ATC161 effectively facilitated p62 oligomerization, a subpopulation of which engaged in the generation of p62 aggregate species (Fig. 2D, lanes 3 ~ 7, and E). ATC161 induced p62 oligomerization at ~ 1 nM in a dose-dependent manner up to 1,000 nM (Fig. 2F and G). Next, we monitored the relative activities of Autotacs to induce the biogenesis of autophagic membranes. Immunoblotting analyses showed that ATC161 (anle138b), ATC163 (resveratrol), and ATC164 (PBA) induced the lipidation of LC3 into LC3-II more efficiently as compared with ATC162 (baicalein) (Fig. 2A and C). ATC161 induced the lipidation of LC3 into LC3-II in a dose-dependent manner up to 1,000 nM (Fig. 2F and H). These results suggest that ATC161 is engaged to p62 and induces p62 polymerization at as low as 1 nM.

It is known that α-syn normally undergoes dynamic structural transitions between monomers and multimers, a subpopulation of which grow into oligomers and fibrils as well as degradation-resistant aggregates during the pathogenesis of PD [57–60]. To determine whether ATC161 exhibits degradative efficacy specifically to α-syn aggregates in comparison with its monomers, SH-SY5Y cells stably expressing A53T mutant α-syn were transduced with α-syn PFFs. The resulting insoluble α-syn aggregates were separated from soluble species using Triton X-100 fractionation assays, followed by immunoblotting analyses. Importantly, ATC161 induced the degradation of α-syn aggregates at a half degradation concentration (DC_50_) of 100 nM (Fig. 2I and K). By sharp contrast, no such degradation was observed for the physiological monomeric species of α-syn in the soluble fraction. Dot blot assays with a 450 nm filter also confirmed that ATC161 induced the degradation of high molecular weight α-syn aggregates at 100 nM (Fig. S3A and B). To further characterize the degradation efficacy of ATC161 in a pathological condition, primary cortex neurons from rat embryonic brains were cultured and subjected to analogous assays, which again showed that ATC161 selectively targets insoluble α-syn species from 10 nM, leaving its soluble monomeric form intact (Fig. 2J and L). These results suggest that ATC161 exhibits degradative efficacy selective to insoluble aggregates of α-syn but not its monomers.

Growing evidence indicates that different species of α-syn spatiotemporally propagate to various brain regions through cell-to-cell transmission [61, 62]. To determine whether the degradation of α-syn aggregates prevents the propagation of α-syn, SH-SY5Y α-syn A53T cells were infected with human *SNCA* overexpression adenovirus and were treated with ATC161, followed by the concentration of extracellular α-syn in the media. ELISA analyses showed that ATC161 reduced the relative level of extracellular total α-syn by 50% (Fig. S3C). Next, to evaluate the propagation of extracellular α-syn into cells, fresh SHSY5Y cells were cultured with these collected media. The level of intracellular α-syn was lower in the total lysates of cells exposed to the collected media from ATC161-treated cells (Fig. S3D and E). These results show that the degradation of α-syn aggregates reduces their transmission into neighboring cells.

To determine whether ATC161 mediates the degradation of α-syn aggregates via macroautophagy, we monitored the metabolic fate of α-syn upon autophagic inhibition using bafilomycin A1. Immunocytochemistry revealed that PFF-induced α-syn aggregates were efficiently eliminated by ATC161 as determined by 55% reduction in the number of α-syn^+^ cytosolic puncta (Fig. 2M and N). ATC161 no longer exhibited such a degradation efficacy when autophagy flux was blocked, confirming the role of autophagy in the activity of ATC161. We then determined whether ATC161 induced the targeting of α-syn aggregates to lysosomes. Immunocytochemistry in SH-SY5Y α-syn A53T cells revealed that the level of LAMP1^+^ lysosomes was attenuated upon PFF-induced α-syn aggregation and readily restored by the blockage of lysosomal degradation (Fig. 2O and P). Despite the quasi-intact autophagic flux toward lysosomes, accumulating α-syn aggregates failed to be targeted to lysosomes (Fig. 2O). Notably, ATC161 significantly increased the level of LAMP1^+^ lysosomes by 2.9-fold, leading to the successful targeting of α-syn aggregates to lysosomes (Fig. 2O-Q). These results demonstrate that ATC161 targets α-syn aggregates to macroautophagy, leading to lysosomal degradation.

### ATC161 targets α-syn aggregates to p62-dependent macroautophagy

**Fig. 3:**
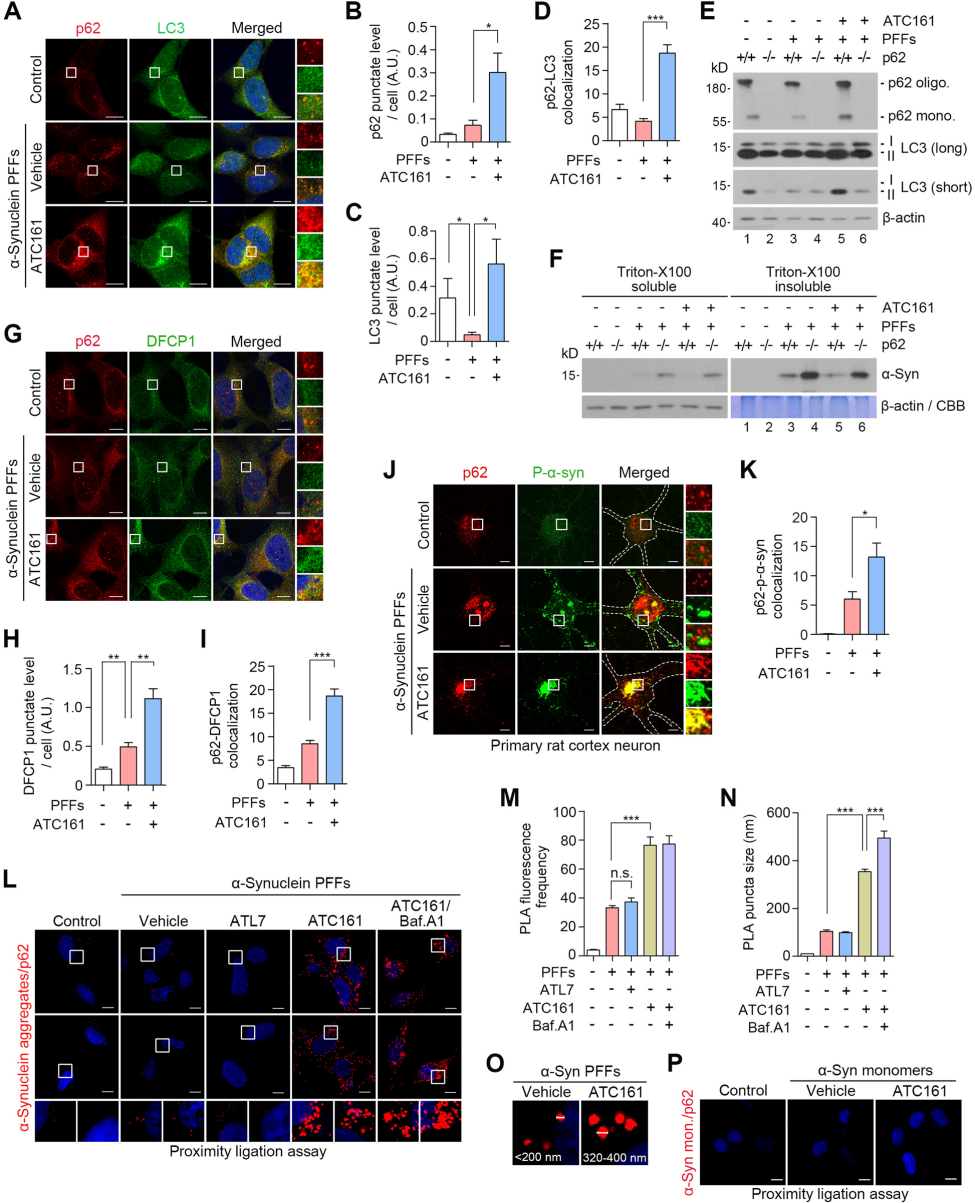
ATC161 targets α-syn aggregates to p62-dependent autophagy. **A** Immunocytochemistry analysis in h-α-syn PFF-transduced HEK293A cells treated with ATC161 (1 μM, 12 h). **B-D** Quantifications of A (*n* = 4–5 for B and C; *n* = 20 cells were counted per group for the number of p62^+^-LC3^+^ colocalizing puncta in D). Data are presented as the mean (SEM). *P*-values (from two-sided unpaired t tests): ^*^ *P* ≤ 0.05, ^***^ *P* < 0.001. **E** Western blot in MEF WT and *p62* knock-out cell-lines. The cells were transduced with h-α-syn PFFs (48 h) and subsequently administered with ATC161 (1 μM, 12 h). **F** Western blot in MEF WT and *p62* knock-out cell-lines transduced with h-α-syn PFFs (48 h) and treated with ATC161 (1 μM, 24 h). The cell lysates were subjected to Triton x-100 fractionation. **G** Immunocytochemistry in HEK293A cells transduced with h-α-syn PFF, followed by ATC161 treatment (1 μM, 12 h). **H** and **I** Quantifications of G (H, *n* = 5; I, *n* = 20 cells counted per group for the number of p62^+^-DFCP1^+^ colocalizing puncta). Data are presented as the mean (SEM). *P*-values (from two-sided unpaired t tests): ^**^ *P*-value < 0.01, ^***^ *P* < 0.001. **J** Immunocytochemistry in rat cortex primary neurons transduced with h-α-syn PFFs, subsequently treated with ATC161 (1 μM, 12 h). **K** Quantification of J (*n* = 12 cells counted per group for the number of p62^+^-p-α-syn^+^ colocalizing puncta). **L** Proximity ligation assay (PLA) in HEK293A cells showing interaction between p62 and α-syn aggregates (with an anti-α-syn aggregate antibody). ATL7 (1 μM, 6 h), ATC161 (1 μM, 6 h), and baf. A1 (200 nM, 6 h) were administered subsequent to h-α-syn PFF transduction (48 h). **M** and **N** Quantifications from L (*n* = 5, *n* = 10, respectively), shown as the mean (SEM). *P*-values (from two-sided unpaired t tests): n.s. (not significant) *P*-value > 0.05, ^***^ *P*-value < 0.001. **O** PLA image from L showing increase in puncta size by ATC161. **P** PLA with an anti-α-syn antibody in HEK293A cells upon transduction with recombinant h-α-syn monomers (48 h) and treatment with ATC161 (1 μM, 6 h). All the scale bars in this figure represent 10 μm

We determined whether ATC161 induces p62-dependent macroautophagy to target α-syn aggregates. Immunocytochemistry of HEK293A cells transduced with α-syn PFFs showed that ATC161 stimulated the formation of p62^+^ as well as LC3^+^ puncta, leading to a marked increase in p62^+^LC3^+^ puncta (Fig. 3A-D), suggesting that ATC161 induced p62-dependent autophagic flux to target p62-cargo complexes to autophagosomes. Next, we determined whether ATC161 exerts its efficacy through p62 using wild-type and *p62* ^−/−^ mouse embryonic fibroblasts (MEFs) transduced with α-syn PFFs. Immunoblotting analyses showed that ATC161 failed to induce the lipidation of LC3 into LC3-II (Fig. 3E, lane 5 vs 6) and the subsequent degradation of α-syn aggregates in the absence of p62 (Fig. 3F, lane 5 vs 6). These results demonstrate that ATC161 targets α-syn aggregates to p62-dependent macroautophagy.

To determine whether ATC161 induces de novo synthesis of autophagic membranes, we monitored the level of newly formed omegasomes. Immunocytochemistry revealed that ATC161 indeed facilitated the biogenesis of omegasomes as determined by the levels of DFCP1^+^ puncta, a marker for early stage autophagic membranes budding from the ER (Fig. 3G and H). Notably, a significant portion of DFCP1^+^ puncta was also positive for p62 (Fig. 3G and I). These results suggest that ATC161-bound p62 migrates to the ER to facilitate neo-synthesis of phagophores. Interestingly, the increased level of DFCP1^+^ puncta upon α-syn aggregation did not correlate to the increased level of LC3^+^ puncta, which implies that the transition from omegasomes into autophagosomes may be the rate-limiting step in α-syn pathology [47].

We also determined whether ATC161 facilitates the interaction between p62 and α-syn aggregates in rat cortex primary neurons. To this end, α-syn aggregates were induced by MPP^+^, 1-methyl-4-phenylpyridinium. Immunocytochemistry showed that ATC161 promoted colocalization between p62 and α-syn aggregates (Fig. S4A and B). It is known that S129-phosphorylated α-syn (p-α-syn) is a dominant species representing approximately 90% of α-syn aggregates [63, 64]. Consistently, immunocytochemistry indeed confirmed that an abundant level of p-α-syn^+^ cytosolic puncta was generated in rat cortex primary neurons transduced with PFFs (Fig. 3J). Notably, ATC161 induced a marked increase in cytosolic puncta doubly positive for p62 and p-α-syn (Fig. 3J and K). Next, we also employed the proximity ligation assay (PLA) that visualizes in situ interactions of proteins within a distance of 40 nm. The PLA with an anti-α-syn aggregate antibody indeed showed that ATC161 dramatically increased the number and sizes of PLA puncta positive for p62 and α-syn aggregates in HEK293A cells transduced with α-syn PFFs (Fig. 3L-O). These PLA^+^ puncta were further enlarged upon autophagic inhibition using bafilomycin A1, indicative of lysosomal degradation (Fig. 3L and N). As opposed to ATC161, the p62 agonist ATL7 failed to enhance the interaction between p62 and α-syn aggregates in PLA (Fig. 3L and M). These results demonstrate that ATC161 induces the engagement of p62 with α-syn aggregates.

To rule out the possibility that ATC161 targets p62 to α-syn monomers, the cells were also transduced with α-syn monomers prepared from exogenously expressed α-syn. ATC161 failed to induce PLA^+^ signals in the cells transduced with α-syn monomers (Fig. 3P), suggesting that anle138b, the TBL moiety of ATC161, selectively targets α-syn aggregates over α-syn monomers.

### ATC161 exhibits therapeutic efficacy in synucleinopathy-associated genotoxicity and mitotoxicity

**Fig. 4:**
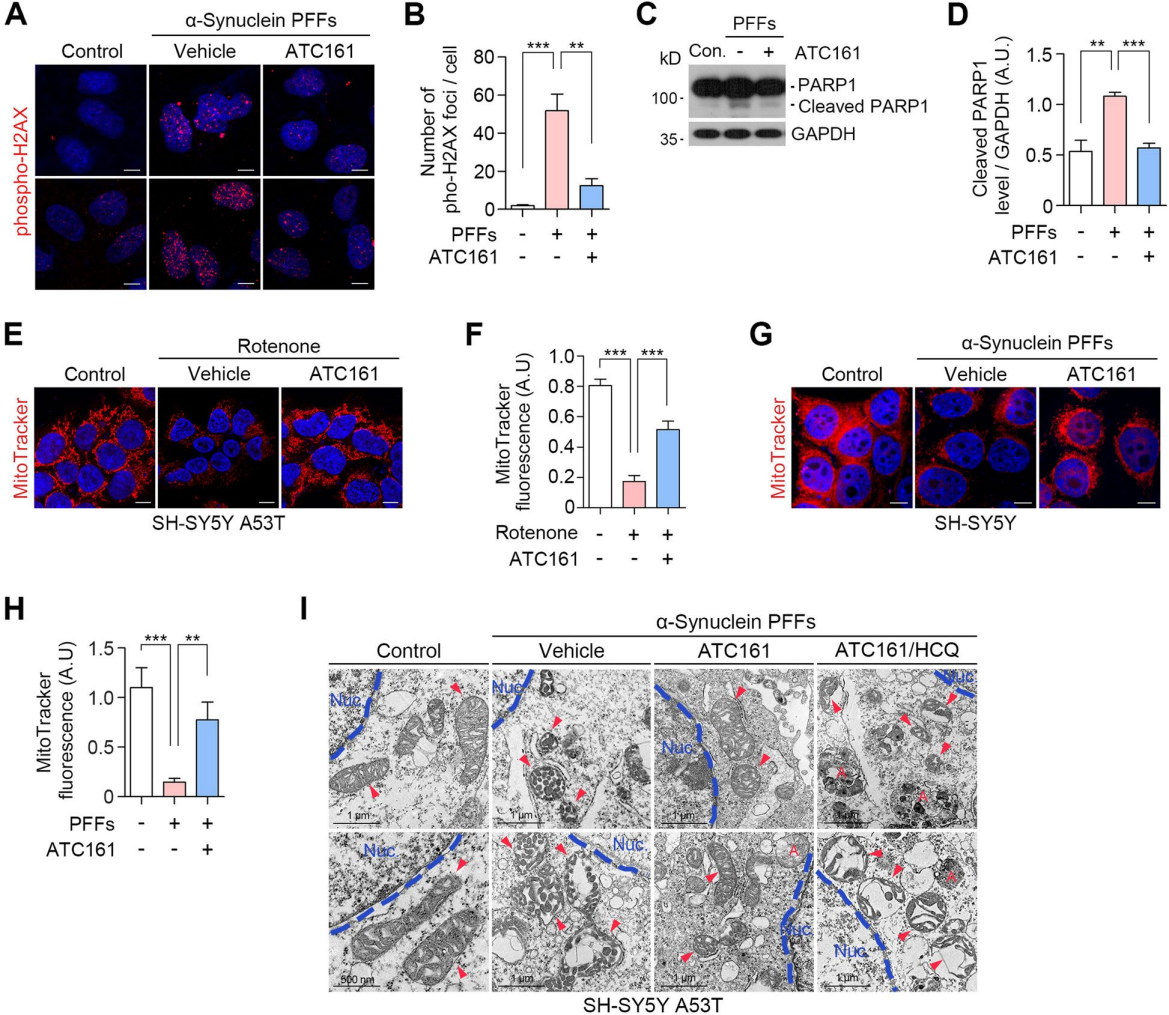
ATC161 ameliorates genotoxicity and mitotoxicity in α-syn pathology. **A** Immunocytochemistry in h-α-syn PFF-transduced HEK293A cells. ATC161 was treated at 0.1 μM for 48 h at 24 h transduction of PFFs. **B** Quantification of A (*n* = 5), the data are presented as the mean (SEM). *P*-values (from two-sided unpaired *t* tests): ^**^ *P*-value < 0.01, ^***^ *P* < 0.001. **C** Western blot in h-α-syn PFF-transduced HEK293A cells (48 h) administered with ATC161 (1 μM, 24 h). **D** Quantification of C (*n* = 3). Data are presented as the mean (SEM). *P*-values (from two-sided unpaired *t* tests): ^**^ *P*-value < 0.01, ^***^ *P* < 0.001. **E** MitoTracker Red analysis in SH-SY5Y α-syn A53T cells. The cells were subjected to rotenone treatment for 48 h followed by ATC161 treatment (0.1 μM, 24 h). **F** Quantification of E (*n* = 7). Data are presented as the mean (SEM) where relevant. *P*-values (from two-sided unpaired *t* tests): ^***^ *P* < 0.001. **G**, MitoTracker Red analysis in wild-type SH-SY5Y cells. The cells were transduced with h-α-syn PFFs (48 h) and subsequently treated with ATC161 (0.1 μM, 24 h). **H**, quantification of G (*n* = 7). Data are presented as the mean (SEM). *P*-values (from two-sided unpaired *t* tests): ^**^ *P*-value < 0.01, ^***^ *P* < 0.001. **I**, transmission electron microscopy analysis in SH-SY5Y α-syn A53T cells transduced with h-α-syn PFF for 24 h prior to treatments. ATC161 (0.1 μM, 48 h) and HCQ (25 μM, 48 h) were treated in the presence of h-α-syn PFFs. Red arrows indicating mitochondria. Letter A in red represents autophagosomes. All the scale bars in this figure represent 10 μm otherwise indicated

Emerging evidence shows that α-syn aggregates induce various cellular implications such as DNA damage associated with DNA double strand breaks (DSBs) [65]. We therefore assessed the efficacy of ATC161 to prevent the formation of DSBs induced by α-syn. Immunocytochemistry revealed that HEK293A cells transduced with α-syn PFFs dramatically accumulated the nuclear foci positive for phospho-H2AX^+^ that marks the sites of DSBs (Fig. 4A and B). Importantly, ATC161 exhibited a robust efficacy to reduce the number of phospho-H2AX^+^ foci (Fig. 4A and B). We therefore monitored the level of PARP1, in which its cleaved form is generated upon apoptotic signaling by excessive DNA damage [66]. As expected, α-syn PFFs increased the levels of cleaved-PARP1 (Fig. 4C and S5A). Notably, ATC161 efficiently inhibited such an apoptotic process (Fig. 4C and D). In addition, when the levels of cleaved-PARP1 were elevated by apoptosis inducers including CCCP and etoposide, no changes were observed by ATC161 (Fig. S5B), indicating that ATC161 exhibits its therapeutic efficacy specific to α-syn pathology. These findings indicate that the degradation of α-syn aggregates by ATC161 inhibits DNA damage and downstream apoptotic signaling during the pathogenesis of PD.

It is known that the accumulation of α-syn causes mitochondrial dysfunction and fragmentation [67]. To determine whether ATC161 protects mitochondria from damage by α-syn, we monitored mitochondrial integrity using MitoTracker Red staining. Fluorescence analyses revealed that α-syn aggregation by rotenone correlated to the diminished level of MitoTracker fluorescence in SH-SY5Y α-syn A53T cells (Fig. 4E and F). No significant changes in the MitoTracker fluorescence were observed when rotenone was treated to wild-type SH-SY5Y cells (Fig. S5C and D). Notably, ATC161 exhibited a significant efficacy in mitochondrial damage, given the increased level of MitoTracker signals as compared to those in vehicle-treated cells (Fig. 4E and F). A similar result was observed in SH-SY5Y cells transduced with α-syn PFFs (Fig. 4G and H). To further validate this finding, we adopted transmission electron microscopy (TEM). As expected, mitochondrial cristae were significantly fragmented and deformed when SH-SY5Y α-syn A53T cells were transduced with α-syn PFFs (Fig. 4I). Importantly, such a damage of mitochondrial cristae was markedly reduced by ATC161. Moreover, ATC161 failed to exert the efficacy when lysosomal degradation was blocked using HCQ as evidenced by mitigated mitochondrial fragmentation and cristae loss (Fig. 4I). These results demonstrate that ATC161 ameliorates, via macroautophagy, the mitotoxicity during the α-syn pathology in PD.

### ATC161 is an orally-administrable drug that ameliorates the pathogenesis in PD mouse model

To determine whether ATC161 exhibits metabolic stability in vivo, we assessed pharmacokinetic profiles upon oral administration in mice. As the p62-targeting moiety of ATC161, ATL13 exhibited high solubility (105.5 μg/ml, pH 7.4) (Fig. S1A) and plasma stability of 96.3% remaining after 120 min (data not shown), which are suitable for drug development. Pharmacokinetic analyses revealed that ATC161 exhibited an effective systemic exposure of 3299.3 ng*h/ml following oral administration (Fig. S1C). The terminal elimination half-life (T_1/2_) of ATC161 in mouse plasma was 3.7 h (Fig. S1C), surpassing the metabolic stability of the aforementioned Autotac compounds (data not shown). Furthermore, ATC161 exhibited approximately 10% brain penetration in TauP301L-BiFC AD mouse model when measured after 4 h of oral administration (Fig. S1C). These suggest that ATC161 is an orally administrable drug.

**Fig. 5:**
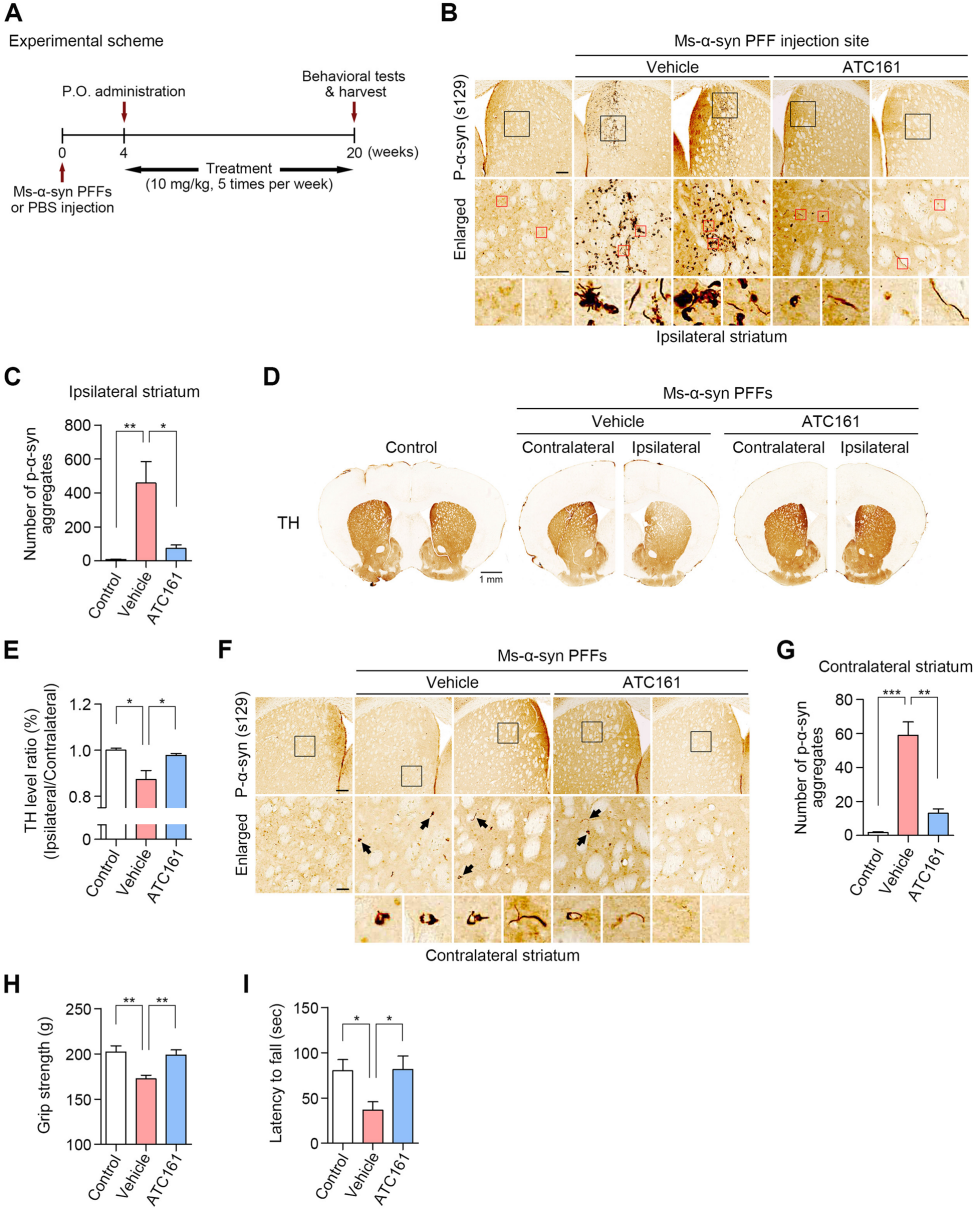
ATC161 is an oral PD drug. **A** Experimental scheme. 5 μg of ms-α-syn PFFs were unilaterally injected into the striatum via stereotaxic surgery, followed by the oral administration of ATC161 at 10 mg/kg for 16 weeks after 4 weeks of the surgery. **B** Immunohistochemistry for p-α-syn^+^ (S129) aggregates in the ms-α-syn PFF-injection site. Scale bars represent 100 μm and 25 μm (for enlarged), respectively. **C** Quantification of B (*n* = 3–4 mice per group). Data are presented as the mean (SEM) where relevant. *P*-values (from two-sided unpaired *t* tests): ^*^ *P* ≤ 0.05, ^**^ *P* < 0.01. **D** TH^+^ (tyrosine hydroxylase) fiber intensity in the striatum. The scale bar represents 1 mm. **E** Quantification of D (*n* = 3–4 mice per group). TH^+^ signal intensity in the PFF injection site (ipsilateral side) was normalized to that of its contralateral side. Data are presented as the mean (SEM) where relevant. *P*-values (from two-sided unpaired *t* tests): ^*^ *P* ≤ 0.05. **F** Immunohistochemistry for p-α-syn^+^ (S129) aggregates in the contralateral striatum. Scale bars represent 100 μm and 25 μm (for enlarged), respectively. **G** Quantification of F (*n* = 3–4 mice per group). Data are presented as the mean (SEM). *P*-values (from two-sided unpaired *t* tests): ^**^ *P*-value < 0.01, ^***^ *P* < 0.001. **H** Grip strength test examining fore- and hindlimb muscle force (*n* = 4–5). **I** Rotarod test examining motor coordination and balance (*n* = 4–5). Data in H and I are presented as the mean (SEM) where relevant. *P*-values (from two-sided unpaired *t* tests): ^*^ *P* ≤ 0.05, ^**^ *P* < 0.01

To test the in vivo therapeutic efficacy of ATC161 in PD, we adopted an α-syn PFF-injected mouse model. C57BL6/J male mice at the age of 8 weeks were unilaterally injected with mouse PFFs into brain striata via stereotaxic surgery. After 4 weeks, the mice were orally administered with vehicle or 10 mg/kg of ATC161 5 times per week for 16 weeks (Fig. 5A). As expected, immunohistochemistry of brain striata revealed that the stereotaxic injection of PFFs induced the accumulation of p-α-syn^+^ signals (S129), in which the large portion formed LB-like inclusions (Fig. 5B). Notably, ATC161 reduced the number of p-α-syn^+^ aggregates in the injection sites compared with those in vehicle-treated mice (Fig. 5B, S6A, and B), indicating that the targeted degradation of p-α-syn^+^ aggregates suppressed the accumulation of LB-like inclusions (Fig. 5C). Accordingly, we determined whether such a degradation efficacy correlates to prevention from the loss of nigrostriatal dopaminergic innervation in the PFF-injected striatum. TH^+^ fiber staining in the ipsilateral striatum showed that the accumulation of p-α-syn^+^ aggregates led to a reduced level of TH^+^ signal (Fig. 5D and E). ATC161 reversed the TH^+^ level to the extent of control group, indicating that the degradation efficacy of ATC161 protected striatal TH^+^ fibers in the PFF-injected striatum (Fig. 5D and E). Next, we determined whether the decreased accumulation of α-syn suppresses its transmission into other brain regions. Immunohistochemistry showed that the stereotaxic injection of PFFs into striata led to the accumulation p-α-syn^+^ signals in the contralateral striata, indicating that pathologic α-syn species were propagated via cell-to-cell transmission (Fig. 5F and G). Importantly, ATC161 attenuated the number of p-α-syn^+^ aggregates in the contralateral striata (Fig. 5F and G). These demonstrate that the suppressed accumulation of α-syn by ATC161 mitigates the transmission of the α-syn aggregate species within mouse brains.

The pathogenesis of synucleinopathy is associated with intense glial response [68]. To determine whether ATC161 ameliorates such immune responses within glial cells, we monitored the level of GFAP^+^ signals, a marker for astrocyte activation. To this end, we performed additional stereotaxic surgery of PFFs directly into mouse brain striata. These mice were subjected to 9 weeks (total 45 treatments) of ATC161 oral administration starting after 3 weeks of the surgery (Fig. S6C). As expected, immunohistochemistry revealed that PFF-injected mice showed a dramatic increase in the number of GFAP^+^ cells in mouse striata near endopiriform nucleus (Fig. S6D and F). Notably, ATC161 reduced the number of GFAP^+^ signals (Fig. S6D and F), along with the decreased number of p-α-syn^+^ aggregates in the corresponding brain area (Fig. S6E and G). This suggests that the reduced number of pathologic α-syn by ATC161 leads to the alleviation of inflammatory response within brain regions.

Next, to determine whether ATC161 prevents the loss of motor functions in the PFF-injected mice, we monitored their grip strength and latency to fall from rotarod in the PD mouse group that received the oral administration of ATC161 for 4 months (Fig. 5A). Grip strength analyses showed that ATC161 increased the peak grip force of fore- and hindlimbs to the extent of control group (Fig. 5H), indicating that the reduction in the muscle force was prevented by ATC161. Consistently, rotarod analyses showed that the mice treated with ATC161 spent more time on rotarod by 59% to the extent of control group, indicating that ATC161 improved motor coordination in the PD mice (Fig. 5I). These suggest that ATC161 exhibits the therapeutic efficacy against the progression of motor deficits in the PD model mice.

## Discussion

Although there have been extensive efforts in developing novel therapeutic approaches for PD, no disease-modifying agents targeting α-syn aggregates are clinically available at present. Here, we developed therapeutic agents that fundamentally degrade α-syn aggregates in PD via the AUTOTAC technology. We designed and synthesized a set of Autotacs and show that ATC161 efficiently induced the degradation of α-syn aggregates via autophagy. Upon targeting α-syn aggregates by the ATC161 TBL, the binding of ATL moiety to p62 induced self-oligomerization of p62 in the complex with aggregates, leading to targeting to LC3^+^ autophagic membranes and lysosomal degradation at 10 nM in PD model cells. Such a degradation was observed with detergent-insoluble α-syn aggregates, but not with functional, monomeric α-syn. Consistently, ATC161 suppressed cell-to-cell transmission of α-syn aggregates in cultured cells. Upon oral administration into PD model mice, ATC161 exhibited therapeutic efficacy to degrade α-syn aggregates in the brains, associated with improvements in glial immune responses and the progression of motor deficits. AUTOTAC is the first TPD demonstrated to selectively degrade α-syn aggregates in vitro and in vivo. Moreover, ATC161, an orally administrable drug, completed its GLP toxicology studies and will be scheduled to enter Phase 1 clinical study, in preparation for Phase 2 studies with PD patients.

The α-syn pathology is implicated by various species including mutant monomeric, oligomeric, and fibrillar forms [11, 58, 69]. Our experimental models were established to generate large α-syn aggregates, raising questions on whether ATC161 targets the other pathogenic α-syn species. Given that the TBL moiety of ATC161, anle138b is known to bind oligomeric signatures within α-syn aggregates, ATC161 is expected to target the oligomeric and fibrillar forms as well. However, aggregation-prone mutants in familial PD such as A53T α-syn was shown to be free from the action of ATC161 (Fig. 2), which can allow the formation of early aggregating species. Future studies can address this for developing Autotacs conjugated with TBLs that target misfolded monomeric α-syn, in which the combination of misfolded α-syn-targeting Autotacs and ATC161 can be synergistic.

In regard to the therapeutic efficacy of ATC161 to alleviate glial inflammatory response (Fig. S6), an interesting question arises on the localization of ATC161. Given that α-syn aggregation occurs in neurons as well as glia and they are both known to induce autophagy upon exposure to protein aggregates [70], we posit that ATC161 can work in both the neuronal and glial cells. In addition, emerging evidence has shown that the glial inflammatory response is triggered by both the extracellular exposure and intracellular accumulation of α-syn [71]. In this regard, we speculate that the alleviated inflammatory response is attributed to the targeted degradation by ATC161 mitigating the propagation of α-syn from neurons as well as reducing the intracellularly accumulated α-syn in glia. It remains interesting to determine whether ATC161 induces therapeutic efficacy specific to cell types. Further studies will address some of these aspects.

Previous studies showed that autophagy can be activated by various chemical inducers such as rapamycin and trehalose in PD models [48, 49, 72]. By inhibiting the mammalian target of rapamycin complex 1 (mTORC1), rapamycin was shown to induce bulk autophagy to promote the clearance of α-syn and neuroprotection [73–75]. However, the mTOR signaling intervenes with biological pathways including inflammation, immunity, and apoptosis [48, 76, 77], limiting its therapeutic potential. Trehalose, a natural disaccharide product that activates the transcription factor EB, also promotes bulk autophagy [72, 78]. Despite the significant activity in autophagy, trehalose failed to reduce the level of high molecular weight α-syn species generated from α-syn PFFs in primary mouse cortex neurons [79]. Consistently, we initially hypothesized that the autophagy induction by p62 agonists could restore neuronal homeostasis from α-syn pathology. Although our data present that the p62 activation upregulates the otherwise suppressed autophagy in our experimental models (Fig. 1), α-syn aggregates were not effectively localized to LC3^+^ autophagic membranes for subsequent degradation. Notably, the chemical conjugation of target-binding ligands to p62 agonists not only effectuated substrate targeting but also sustained the autophagy-inducing activity for the lysosomal degradation. These suggest that the mere activation of macroautophagy is not sufficient to clear α-syn aggregates, perhaps owing to the lack of cargo selectivity. Moreover, these imply that both the target-binding and autophagy-inducing activities are essential for an effective TPD technology in PD.

Our previous work has established the foundation for developing p62 ZZ-domain binding ligands, leading to target sequestration when constituted in Autotacs. Of note, the sequestration mode of action spatially concentrates pathologic substances, which in turn can reduce potential cellular damage. More importantly, we posit that such a p62 activation plays pivotal roles in overcoming the autophagy downregulation in α-syn implicated models. Given the evidence that the α-syn overexpression cellular model showed decreased Rab1a activity with subsequent reduction in the activity of Atg9, causing decreased DFCP1^+^ and LC3^+^ punctate levels [46], we speculated that high molecular weight toxic α-syn species are likely to be involved in the suppression of autophagosome formation. This finding was consistent with our data in PFF-seeded cells, in which the level of LC3^+^ puncta was significantly suppressed (Figs. 1 and 2). The increase in the DFCP1^+^ punctate level in the presence of PFFs did not coincide with the increased level of LC3^+^ puncta, which demonstrates that the transition from omegasomes into autophagosomes is a rate-limiting step that triggers the suppressed autophagosome biogenesis in our experimental model (Fig. 3). However, ATC161 increased the localization of p62 puncta to ER-derived omegasomes, which corresponded with the increased level of LC3^+^ puncta (Fig. 3). This suggests that the action of ATC161 bypasses the downregulation of autophagosome biogenesis in the α-syn pathology. The mechanistic aspect of p62 activation taking a detour from such suppression seems to involve the induction of selective autophagy, as several lines of evidence indicated that p62 oligomers with their cargo substrates are directly localized to FIP200 for autophagosome formation [80, 81].

The accumulation of α-syn was previously shown to cause lysosomal dysfunction [82]. Consistently, we show that α-syn PFFs reduced the level of LAMP1^+^ puncta, in which the p62 activation by ATC161 significantly upregulated the level of LAMP1 (Fig. 2). A distinct study with mouse hearts indicated that p62 exerts a feedforward effect on the activation of TFEB upon proteasomal inhibition [83], by which the activated TFEB can induce lysosome biogenesis. Given that α-syn oligomeric and fibrillar species induce proteasomal malfunction [84], we speculate that the p62 activation by ATC161 leading to lysosomal upregulation in the PD model cells may correlate with such a pathway. Although there are still interesting questions yet to be answered on the precise mechanisms of Autotacs reversing autophagy in the otherwise compromised conditions, our results suggest that ATC161 induces autophagy for targeting α-syn aggregates while overcoming the lysosomal perturbations in PD.

As the number of individuals with PD has increased globally in the last decades [85], it is increasingly urgent to develop novel therapeutic strategies that are fundamentally different from conventional drugs. Although targeting α-syn aggregates has drawn attention as the feasible approach, bulk autophagy inducers were shown to lack selectivity, limiting its therapeutic application. Accordingly, TPD technologies were utilized to develop substrate-specific degraders. However, the TPDs harnessing CMA and UPS are inevitably restrained from degrading α-syn aggregate species due to size limitations. This has necessitated the development of autophagy-dependent TPD, in which the AUTOTAC technology serves as an applicable platform. Moreover, an effective therapeutic strategy should also enable the restoration from autophagy downregulation implicated in PD. These emphasize the clinical significance of Autotacs as the first reported TPD therapeutics for α-syn aggregates.

## Conclusions

Our study demonstrates that the AUTOTAC platform enables the development of target degraders for α-syn aggregates as a novel disease-modifying therapeutic strategy for PD. Where the autophagy-enhancing and the aggregate-targeting activities are significant to develop a successful TPD strategy in PD, the p62-activating and target-binding efficacy of ATC161 effectively degrades α-syn aggregates in PD model cells and mice. This study sheds light on the future clinical strategies for patients with PD as the first to report macroautophagy-dependent TPD.

## Materials and methods

### Cell culture

Wild type human neuroblastoma cells (SH-SY5Y) and Hela were obtained from Korean Cell Line Bank. SH-SY5Y α-syn A53T cells, COS7 cells and 293A cell line were kindly gifted from S.J.L. lab. COS7 cells, 293A cells, wild-type and *p62*^*−/−*^ MEF cells, and wild type or A53T mutant α-syn SH-SY5Y cells were maintained in Dulbecco’s Modified Eagle’s Medium (DMEM) containing 10% fetal bovine serum (FBS). Wild-type and *p62*^−/−^ MEFs were obtained from Keiji Tanaka’s laboratory (Tokyo Metropolitan Research Institute, Japan). Cells were incubated at 37 °C in a 5% CO_2_ humidified chamber. The differentiation of SH-SY5Y α-syn A53T cells was done by 10 µM retinoic acid for 7 days prior to subsequent experiments.

### Antibodies

The followings are primary antibodies used: mouse monoclonal anti-SQSTM1/p62 (Abcam, ab56416; 1:20,000 for WB, 1:300 for ICC), rabbit polyclonal anti-LC3 (Sigma-Aldrich, L7543; 1:10,000 for WB, 1:300 for ICC), rabbit polyclonal anti-LAMP1 (Sigma-Aldrich, L1418; 1:200 for ICC), rabbit polyclonal anti-GAPDH (BioWorld, AP0066; 1:5,000 for WB), rabbit polyclonal anti-β-actin (BioWorld, AP0060; 1:5,000 for WB), mouse monoclonal anti-α-synuclein (BD Transduction, 610,787; 1:2000 for WB, 1:300 for ICC), rabbit monoclonal anti-α-synuclein (phospho-S129) (Abcam, ab51253; 1:200 for ICC, 1:500 for IHC), mouse monoclonal anti-c-Myc (Santa Cruz Biotechnology, H1721; 1:2,000 for WB) rabbit polyclonal anti-DFCP1/ZFYVE1 (ABclonal, A7527; 1:150 for ICC), rabbit monoclonal anti-PARP (Cell Signaling Technology, 9532; 1:1,000 for WB), rabbit monoclonal anti-GFAP (Cell Signaling Technology, D1F4Q; 1:500 for IHC), and mouse monoclonal anti-phospho-Histone H2A.X (ser139) (Upstate, 05–636; 1:200 for ICC). The followings are secondary antibodies used: anti-rabbit IgG-HRP (Cell Signaling Technology, 7074; 1:5,000), anti-mouse IgG-HRP (Cell Signaling Technology, 7076; 1: 5,000), Alexa fluor 488 goat anti-rabbit IgG (A11008; 1:500), and Alexa fluor 555 goat anti-mouse IgG (Invitrogen, A11029; 1:500).

### Primary neuron culture

All procedures were conducted according to the IACUC and were approved by Seoul National University Institutional Animal Care and Use Committee. Rat cortex primary neuron were prepared from E16 Sprague–Dawley rat embryos (Orient Bio). Anesthetized mice were sacrificed by cervical dislocation, and embryos were removed. Cortices were dissected on the ice. Neurons were trypsinized, dissociated and plated onto poly-d-lysine coated cover slips at a density of 5 × 10^4^ cells per cm^2^ per 12-well plate or 6-well plate. Primary neurons were maintained in Neurobasal A medium containing 2% B27, 0.5 mM glutamax and antibiotics. Cells at 7 DIV were treated with h-α-syn PFF (1 µg/mL) for 14 d.

### In vitro fibrillization of alpha-synuclein and treatment

Recombinant human α-syn (1–140) were obtained from NKMAX (SNA2001L). In vitro fibrillization was performed by diluting recombinant α-syn monomers to 1 mg/mL in sterile PBS, then incubating at 37 °C with constant agitation at 1,000 rpm for seven days. The fibrillization was verified by thioflavin T and sedimentation assays. The ability to form phospho-α-syn (S129) was also assessed by immunocytochemistry. In addition, to determine whether the generated PFFs act as seeds to accumulate endogenous α-syn, an adenovirus that expresses c-Myc-His-tagged human α-syn (gifted from lab of S.J.L) was used to infect cultured cells at an MOI of 10, followed by a fractionation assay. The administration of PFFs into 293A cells was done with protein delivery reagent (BioPORTER QuikEase Protein Delivery Kit, Sigma) following the provider’s instruction. The mouse α-syn PFFs were obtained from StressMarq (SPR-324). Prior to treatment, PFFs were sonicated at 20% amplitude, 1 s pulse on and off for 30 s (SONICS Vibra Cell sonicator, Merck Z412619). For cells, α-syn monomers and PFFs were transduced at 1 µg/mL.

### Rotenone-induced cellular PD model

Adenovirus overexpressing human wild-type *SNCA* was kindly gifted from the lab of S.J.L. COS7 cells were infected with the adenovirus overexpressing human α-syn at 0.8 × 10^8 pfu and incubated at 37 °C for 24 h. When the confluency reached 90%, the cells were split onto 6-well plates for further experiments. The α-syn aggregation was induced by the treatment of 100 nM rotenone (Sigma-Aldrich, R8875) for 56 h. For the MitoTracker analyses in SH-SY5Y α-syn A53T cells, rotenone was treated at 1 µM for 48 h with the co-treatment of 100 nM ATC161 for 24 h, then MitoTracker Red CMXRos (Invitrogen; M7512) was used to stain intact mitochondria following the manufacturer’s instruction.

### Immunocytochemistry

Cells were fixed with 4% paraformaldehyde (PFA) for 20 min at room temperature, followed by permeabilization with 0.1% Triton X-100 buffer for 15 min. The cells were blocked with 2% bovine serum albumin (BSA) in PBS for 1 h at room temperature. Then, the cells were incubated with primary antibodies diluted in the blocking buffer overnight at 4 °C. After washing with PBS, the slides were incubated with AlexaFluor-conjugated secondary antibodies for 1 h at room temperature. After mounting the slides with mounting solution containing DAPI, the images were captured on an LSM700 confocal microscopy (Carl Zeiss).

### Immunoblotting

For the separation of Triton X-100 soluble and insoluble fractions for monitoring α-syn, cells were washed with PBS and lysed in Triton X-100 buffer (50 mM Tris / 150 mM NaCl / 100 mM EDTA / 1% Triton X-100, pH 7.6) containing protease and phosphatase inhibitors for 30 min on ice. The lysates were centrifuged at 12,000 rpm for 30 min, and the soluble supernatant was taken. The remaining pellets were washed with Triton X-100 buffer, followed by centrifugation at 12,000 rpm for 20 min. The supernatants were discarded, and the resulting insoluble pellets were resuspended in laemli buffer containing 1% SDS. Samples were subjected to SDS-PAGE. After blocking with 5% milk and washing process, the membranes were incubated with primary antibodies overnight at 4 °C, followed by washing with PBS-T and incubation with horseradish peroxidase-conjugated secondary antibodies. Immunoreactive bands were detected using ECL reagents (Thermo Fisher Scientific, 32,106). For non-reducing SDS-PAGE, cell lysates were mixed with 4 × laemli buffer containing no 2-mercaptoethanol. For reducing SDS-PAGE, cells were lysed by RIPA buffer (150 mM NaCl, 1% Triton X-100, 1% sodium deoxycholate, 0.1% Tris–HCl, pH 7.5, 2 mM EDTA) and prepared with 5 × protein sample buffer containing 2-mercaptoethanol (Elpis Biotech, EBA-1052). Densitometry of developed bands was analyzed with ImageJ (NIH, Bethesda).

### Proximity ligation assay

Proximity ligation assays were conducted using a PLA kit (Sigma-Aldrich) according to the manufacturer’s protocol. 293A Cells were seeded on coverslips and cultured for 24 h. After fixation with 4% PFA, the cells were permeabilized with 0.1% Triton X-100. The cells were incubated with antibodies for p62 (Abcam; ab56319) and alpha-synuclein aggregates (Abcam; ab209538) overnight at 4 °C. After washing with PLA washing buffer, the cells were hybridized, ligated and amplified by rolling circle. Coverslips were mounted on slides using mounting medium containing DAPI, and the images were captured by confocal microscopy (Zeiss).

### In vitro p62 aggregation assay

HEK293 cells were lysed by 10 freeze–thaw cycles in lysis buffer (50 mM HEPES, pH 7.4, 0.15 M KCL, 0.1% Nonidet P-40, 10% glycerol, and a mixture of protease and phosphatase inhibitors), followed by centrifugation at 13,000 × g. The protein concentration of the supernatant was determined using BCA assay. The p62-Flag plasmid synthesized in our previous study was transfected into the 293 cells. After cell lysis, total of 5 µg protein was mixed with ATLs at the final concentration of 1 mM in the final volume of 20 µL. These samples were incubated for 2 h at room temperature. The samples were then prepared with non-reducing sampling buffer containing 4% lithium dodecyl sulfate and boiled at 95 °C for 10 min, and then subjected to SDS-PAGE in a gradient gel and immunoblotting.

### Conditioned media collection and treatment

The conditioned medium was collected from differentiated SH-SY5Y cells infected with adenovirus overexpressing human α-syn. Following 24 h infection, the medium was replaced with fresh media containing DMSO or ATC161. The medium was collected after 24 h and centrifuged at 1,000 rpm to remove cell debris and dead cells. The supernatant was concentrated using Amicon 10 K MWCO filters (Millipore, Billerica, MA). The amounts of α-syn in conditioned medium were measured by enzyme-linked immunosorbent assay (ELISA) with an ELISA kit (Abcam, human α-syn ELISA kit, ab260052) following the provider’s instruction. The subsets of collected media were treated to fresh SH-SY5Y cells in order to determine the level of α-syn propagated to the fresh cells.

### Quantification of TH in striatal area

Mice were anesthetized and perfused with PBS and 4% paraformaldehyde. Brains were sectioned at 30 μm thickness by vibratome (Leica). Free-floating sections were blocked with PBS containing 5% serum and 0.3% Triton X-100 for 1 h. The sections were then incubated with an anti-tyrosine hydroxylase (TH) antibody (1:5,000, Sigma, T1299) overnight at 4℃. The sections were incubated with biotinylated secondary antibody for 1 h at room temperature. The signal amplification was achieved by the ABC kit (Vector) followed by development with DAB (Vector). TH^+^ optical density was measured in three brain sections per animal. Images of the ipsilateral and contralateral striatal area were obtained using an Axio scan Z1 slide scanner (Zeiss). Images were converted to grayscale via Image J, and the intensity was analyzed. Normalized values across the sections were used to compare the relative TH^+^ optical density.

### Transmission electron microscopy (TEM)

SH-SY5Y cells were trypsinized and pelleted by centrifugation. The pellets were fixed with 0.5% glutaraldehyde for overnight at 4 °C. Ultra-thin section of the pellets was prepared with ultramicrotome (Leica). Images were captured on TEM electron microscope JEM-1400 at Electron Microscopy Center, Seoul National University Hospital Biomedical Research Institute.

### Intrastriatal injection of mouse α-synuclein pre-formed fibrils and drug administration

Intrastriatal injection of α-syn fibrils was performed in C57BL6/J male mice at 8 weeks of age. 5 µg of α-syn fibrils were sonicated and injected into the dorsal striata in mice at the empirically derived coordinates: 1.0 mm anterior and 1.5 mm lateral to the Bregma, and 3.0 mm ventral relative to the skull in the course of 20 min. Scalp incisions were closed by suture. Three or four weeks later, the mice were orally treated with 10 mg/kg of ATC161 5 times per week for 9 or 20 weeks.

### Filter trap assay for α-synuclein aggregates

Differentiated SH-SY5Y α-syn A53T cells were treated with 2 μg/ml of a-syn PFFs, and after 24 h cells were treated with 100 nM of ATC161 for 24 h. The cells were lysed in RIPA buffer for 30 min on ice and then centrifuged at 13,000 rpm for 20 min at 4 ℃. The supernatants were aspirated and transferred into a new tube kept on ice. Protein concentration was determined by a BCA assay. The filter trap assay was performed with 5 μg of total protein diluted in 100 μL of lysate. A nitrocellulose membrane (pore size: 0.45 μm) was placed on the filter trap apparatus (96-well Bio-Dot®). Lysates were loaded and allowed to pass through the membrane under vacuum. Aggregated α-syn levels were determined by immunoblotting using an anti-α-syn aggregate antibody [MJFF-14–6-4–2] (1:2000; Abcam, ab209538).

### Immunohistochemistry

Brain sections were cut into 30 µm on a freezing microtome, and stored in tissue storage solution at -20 °C. Sections were washed with PBS and incubated with blocking buffer (2% normal goat serum, 1% BSA, 1% Triton X-100, and Tween-20 in PBS) for 1 h at room temperature. After washing with PBS, sections were incubated with primary antibodies (pS129-α-syn, GFAP) overnight at 4 °C, followed by secondary Goat anti-mouse AlexaFluor488 and Goat anti-rabbit AlexaFluor555 for 1 h at room temperature. Images were processed by Axio Scan Z1 (Carl Zeiss). In order to quantify the number of neurons in the PFF-injected striatum, the brain sections were stained with 0.1% crystal violet solution containing 0.1% acetic acid for 1 min at 50 ℃ and then rinsed in distilled water. Sections were dehydrated in 95% and 100% alcohol and mounted on slides.

### Behavioral test

The behavioral tests were performed according to IACUC approval (AUTOTAC Bio Inc., ATB-2107–06-1). The previously generated PFF-injected mouse model was used. For the forelimb grip strength test, a grip strength meter (Bioseb, BIO-GS3) was used. Three consecutive measurements were done for each mouse, and the average was calculated. For the motor coordination test, a rotarod (Bioseb, BX-ROD) was used. Each trial consisted of three attempts. The rotating speed was gradually increased from 4 to 40 rpm in the total time of 300 s.

### ATL and Autotac compounds

The chemical syntheses of Nt-Arg-mimicking and Autotac compounds are described in the Supplementary Methods.

### Statistical analysis

The data are presented as the mean (± SEM). For data analysis, GraphPad Prism 8 software was used. Statistics were performed using two-tailed unpaired Student’s t-test. Values were considered significant for *p < 0.05, **p < 0.01, and ***p < 0.001.

### Supplementary Information

**Additional file 1: Supplementary figure 1.** Drug profiles of ATL and Autotac compounds.** A**, drug profiles of ATL compounds. Tissue concentrations and oral bioavailability were measured in ICR male mice upon 5 mg/kg oral administration of each ATL compound. **B**, structures of ATC161~164. **C**, Pharmacokinetic study of ATC161 conducted in male ICR mice otherwise indicated. M.W. represents molecular weight. The plasma exposure and the half-life were determined upon either POor IPATC161 administration. The drug tissue exposure was determined in Tau301L BiFC mice. IV refers to intravenous injection.

**Additional file 2: Supplementary figure 2.** ATLs induce autophagy but fail to degrade α-syn aggregates.** A**, western blot of c-Myc in HEK293A cells infected with c-Myc-His-tagged human α-syn expressing adenovirus. The adenovirus was infected at an MOI of 10 for 24 h and PFFs were transduced for 48 h subsequently. The cells were subjected to Triton X-100 fractionation assay to determine whether the PFFs act as seeds to accumulate endogenously expressed c-Myc-α-syn in the insoluble fraction. **B**, western blot in COS7 cells infected with h-α-syn overexpressing adenovirus followed by rotenone treatment. ATL compounds were treated at 1 μM for 24 h. **C**, western blot in the Triton x-100 insoluble fraction of the COS7 cells.

**Additional file 3: Supplementary figure 3.** ATC161 induces targeted degradation of HMW α-syn aggregates and reduces cell-to-cell transmission of α-syn. **A**, dot blot assay in α-syn A53T SH-SY5Y cells differentiated by RA. 2 μg/mL of PFFs were transduced for 48 h followed by ATC161 treatmentfor 24 h. **B**, quantification of A. Data are presented as the meanwhere relevant. *P*-values: ^**^P-value < 0.01. **C**, ELISA of the conditioned media from cultured SH-SY5Y α-syn A53T cells infected with h-α-syn overexpressing adenovirus, followed by treatment with ATC161. Data are presented as the meanwhere relevant. *P*-values: ^*^P ≤ 0.05, ^**^P-value < 0.01. **D**, western blot in fresh SH-SY5Y cells cultured with the conditioned media from C. **E**, quantification of D. Data are presented as the meanwhere relevant. *P*-value: ^*^P ≤ 0.05.

**Additional file 4: Supplementary figure 4.** ATC161 targets α-syn aggregates to p62 in MPP^+^-modeled primary neurons. **A**, immunocytochemistry in rat cortex primary neurons treated with 5 mM MPP^+^ for 6 h, followed by ATC161 treatment at 1 μM for 3 h. Scale bars represent 10 μm. **B**, quantification of A. The numbers of α-syn inclusions positive for P62^+^ puncta were counted per cell. Data are presented as the meanwhere relevant. *P*-value: ^***^P < 0.001.

**Additional file 5: Supplementary figure 5.** ATC161 exhibits therapeutic efficacy specific to α-syn pathology. **A**, western blot of PARP1 in HEK293A cells subsequent to HCQ treatment at 25 μM for 24 h. Since HCQ itself induces the formation of cleaved PARP1, the use of HCQ cannot determine the autophagy-dependency of therapeutic efficacy in PFF-transduced cells. **B**, western blot of PARP1 in HEK293A cells. The cells were treated with either CCCPor etoposidefor 24 h followed by ATC161 treatment at 1 μM for 24 h. **C**, MitoTracker Red analyses in wild-type SH-SY5Y cells co-treated with rotenone at 1 µM and ATC161 at 1 µM for 24 h. **D**, quantification of C. *P*-values: n.s.*P*-value > 0.05. Scale bars represent 10 μm.

**Additional file 6: Supplementary figure 6.** ATC161 reduces glial inflammatory response in PD mice. **A**, Nissl staining in the PFF stereotaxic surgery injection site. The ATC161 oral administration did not induce neuronal cell death at 10 mg/kg. **B**, quantification of A. *P*-values: n.s.*P*-value > 0.05. **C**, experimental scheme for mice subjected to ATC161 oral administration at 10 mg/kg for 9 weeks. The mice were subjected to stereotaxic injection of PFFsand were administered with the drug after 3 weeks of the surgery. **D**, immunohistochemistry of the mouse brain sections for the GFAP^+^ signal in endopiriform nucleus. Scale bars represent 500 μm and 25 μm, respectively. **E**, immunohistochemistry of the mouse brain sections for p-α-syn^+^aggregates in endopiriform nucleus. Scale bars represent 50 μm. **F** and **G**, quantifications of D and E, respectively. Data are presented as the meanwhere relevant. *P*-values: ^*^P ≤ 0.05.

**Additional file 7. **Supplementary Methods.

## Data Availability

All data are available within the published article and its supplementary files.

## Abbreviations

PD: Parkinson’s disease
LB: Lewy body
α-Syn: Alpha-synuclein
TPD: Targeted protein degradation
AUTOTAC: Autophagy targeting chimera
PFF: Preformed-fibril
TBL: Target binding ligand
ATL: Autophagy targeting ligand
UPS: Ubiquitin-proteasome system
CMA: Chaperone-mediated autophagy
PROTAC: Proteolysis-targeting chimera
UBR1 to 4: E3 ubiquitin-protein ligase UBR1 to 4
Arg: Arginine
HCQ: Hydroxychloroquine
Baf. A1: Bafilomycin A1
WB: Western blot
ICC: Immunocytochemistry
IHC: Immunohistochemistry
PLA: Proximity ligation assay
ELISA: Enzyme-linked immunosorbent assay
K_d_: Dissociation constant
P62/SQSTM1: Sequestosome 1
LC3: Microtubule-associated protein 1A/1B-light chain 3
GAPDH: Glyceraldehyde-3-phosphate dehydrogenase
DFCP1: Double FYVE-containing protein 1
LAMP1: Lysosomal-associated membrane protein 1
PARP: Poly (ADP-ribose) polymerase

## References

[1] Mhyre TR, Boyd JT, Hamill RW, Maguire-Zeiss KA (2012). Parkinson's disease. Subcell Biochem.

[2] Poewe W, Seppi K, Tanner CM, Halliday GM, Brundin P, Volkmann J, Schrag AE, Lang AE (2017). Parkinson disease. Nat Rev Dis Primers.

[3] Luk KC, Kehm V, Carroll J, Zhang B, O'Brien P, Trojanowski JQ, Lee VM (2012). Pathological α-synuclein transmission initiates Parkinson-like neurodegeneration in nontransgenic mice. Science.

[4] Gómez-Benito M, Granado N, García-Sanz P, Michel A, Dumoulin M, Moratalla R (2020). Modeling Parkinson's Disease With the Alpha-Synuclein Protein. Front Pharmacol.

[5] Wan OW, Chung KK (2012). The role of alpha-synuclein oligomerization and aggregation in cellular and animal models of Parkinson’s disease. PLoS ONE.

[6] Ghosh D, Mehra S, Sahay S, Singh PK, Maji SK (2017). α-synuclein aggregation and its modulation. Int J Biol Macromol.

[7] Manning-Bog AB, McCormack AL, Li J, Uversky VN, Fink AL, Di Monte DA (2002). The herbicide paraquat causes up-regulation and aggregation of alpha-synuclein in mice: paraquat and alpha-synuclein. J Biol Chem.

[8] Betarbet R, Sherer TB, MacKenzie G, Garcia-Osuna M, Panov AV, Greenamyre JT (2000). Chronic systemic pesticide exposure reproduces features of Parkinson's disease. Nat Neurosci.

[9] Brown TP, Rumsby PC, Capleton AC, Rushton L, Levy LS (2006). Pesticides and Parkinson’s disease–is there a link?. Environ Health Perspect.

[10] Cascella R, Bigi A, Cremades N, Cecchi C (2022). Effects of oligomer toxicity, fibril toxicity and fibril spreading in synucleinopathies. Cell Mol Life Sci.

[11] Mahul-Mellier AL, Burtscher J, Maharjan N, Weerens L, Croisier M, Kuttler F, Leleu M, Knott GW, Lashuel HA (2020). The process of Lewy body formation, rather than simply α-synuclein fibrillization, is one of the major drivers of neurodegeneration. Proc Natl Acad Sci USA.

[12] Hayes MT (2019). Parkinson’s Disease and Parkinsonism. Am J Med.

[13] Armstrong MJ, Okun MS (2020). Diagnosis and treatment of Parkinson disease: a review. JAMA.

[14] Dong J, Cui Y, Li S, Le W (2016). Current pharmaceutical treatments and alternative therapies of Parkinson’s disease. Curr Neuropharmacol.

[15] Fox SH, Katzenschlager R, Lim SY, Barton B, de Bie R, Seppi K, Coelho M, Sampaio C, Movement Disorder Society Evidence-Based Medicine Committee (2018). International Parkinson and movement disorder society evidence-based medicine review: Update on treatments for the motor symptoms of Parkinson’s disease. Mov Disord.

[16] Xilouri M, Brekk OR, Stefanis L (2013). α-Synuclein and protein degradation systems: a reciprocal relationship. Mol Neurobiol.

[17] Machiya Y, Hara S, Arawaka S, Fukushima S, Sato H, Sakamoto M, Koyama S, Kato T (2010). Phosphorylated alpha-synuclein at Ser-129 is targeted to the proteasome pathway in a ubiquitin-independent manner. J Biol Chem.

[18] Emmanouilidou E, Stefanis L, Vekrellis K (2010). Cell-produced alpha-synuclein oligomers are targeted to, and impair, the 26S proteasome. Neurobiol Aging.

[19] Lee HJ, Khoshaghideh F, Patel S, Lee SJ (2004). Clearance of alpha-synuclein oligomeric intermediates via the lysosomal degradation pathway. J Neurosci.

[20] Cuervo AM, Stefanis L, Fredenburg R, Lansbury PT, Sulzer D (2004). Impaired degradation of mutant alpha-synuclein by chaperone-mediated autophagy. Science.

[21] Martinez-Vicente M, Talloczy Z, Kaushik S, Massey AC, Mazzulli J, Mosharov EV, Hodara R, Fredenburg R, Wu DC, Follenzi A, Dauer W, Przedborski S, Ischiropoulos H, Lansbury PT, Sulzer D, Cuervo AM (2008). Dopamine-modified alpha-synuclein blocks chaperone-mediated autophagy. J Clin Investig.

[22] Watanabe Y, Tatebe H, Taguchi K, Endo Y, Tokuda T, Mizuno T, Nakagawa M, Tanaka M (2012). p62/SQSTM1-dependent autophagy of Lewy body-like α-synuclein inclusions. PLoS ONE.

[23] Odagiri S, Tanji K, Mori F, Kakita A, Takahashi H, Wakabayashi K (2012). Autophagic adapter protein NBR1 is localized in Lewy bodies and glial cytoplasmic inclusions and is involved in aggregate formation in α-synucleinopathy. Acta Neuropathol.

[24] Schlossmacher MG, Frosch MP, Gai WP, Medina M, Sharma N, Forno L, Ochiishi T, Shimura H, Sharon R, Hattori N, Langston JW, Mizuno Y, Hyman BT, Selkoe DJ, Kosik KS (2002). Parkin localizes to the Lewy bodies of Parkinson disease and dementia with Lewy bodies. Am J Pathol.

[25] Kuusisto E, Parkkinen L, Alafuzoff I (2003). Morphogenesis of Lewy bodies: dissimilar incorporation of alpha-synuclein, ubiquitin, and p62. J Neuropathol Exp Neurol.

[26] Bohush A, Niewiadomska G, Weis S, Filipek A (2019). HSP90 and Its Novel Co-Chaperones, SGT1 and CHP-1, in Brain of Patients with Parkinson's Disease and Dementia with Lewy Bodies. J Parkinsons Dis.

[27] Zhao L, Zhao J, Zhong K, Tong A, Jia D (2022). Targeted protein degradation: mechanisms, strategies and application. Signal Transduct Target Ther.

[28] Fan X, Jin WY, Lu J, Wang J, Wang YT (2014). Rapid and reversible knockdown of endogenous proteins by peptide-directed lysosomal degradation. Nat Neurosci.

[29] Qu J, Ren X, Xue F, He Y, Zhang R, Zheng Y, Huang H, Wang W, Zhang J (2020). Specific Knockdown of α-Synuclein by Peptide-Directed Proteasome Degradation Rescued Its Associated Neurotoxicity. Cell Chem Biol.

[30] Kargbo RB (2020). PROTAC Compounds Targeting α-Synuclein Protein for Treating Neurogenerative Disorders: Alzheimer's and Parkinson's Diseases. ACS Med Chem Lett.

[31] Hinault MP, Cuendet AF, Mattoo RU, Mensi M, Dietler G, Lashuel HA, Goloubinoff P (2010). Stable alpha-synuclein oligomers strongly inhibit chaperone activity of the Hsp70 system by weak interactions with J-domain co-chaperones. J Biol Chem.

[32] Rubin DM, Finley D (1995). Proteolysis. The proteasome: a protein-degrading organelle?. Curr Biol.

[33] Sriram SM, Kim BY, Kwon YT (2011). The N-end rule pathway: emerging functions and molecular principles of substrate recognition. Nat Rev Mol Cell Biol.

[34] Kwon YT, Xia Z, Davydov IV, Lecker SH, Varshavsky A (2001). Construction and analysis of mouse strains lacking the ubiquitin ligase UBR1 (E3alpha) of the N-end rule pathway. Mol Cell Biol.

[35] Tasaki T, Mulder LC, Iwamatsu A, Lee MJ, Davydov IV, Varshavsky A, Muesing M, Kwon YT (2005). A family of mammalian E3 ubiquitin ligases that contain the UBR box motif and recognize N-degrons. Mol Cell Biol.

[36] Sriram SM, Banerjee R, Kane RS, Kwon YT (2009). Multivalency-assisted control of intracellular signaling pathways: application for ubiquitin- dependent N-end rule pathway. Chem Biol.

[37] Cha-Molstad H, Sung KS, Hwang J, Kim KA, Yu JE, Yoo YD, Jang JM, Han DH, Molstad M, Kim JG, Lee YJ, Zakrzewska A, Kim SH, Kim ST, Kim SY, Lee HG, Soung NK, Ahn JS, Ciechanover A, Kim BY (2015). Amino-terminal arginylation targets endoplasmic reticulum chaperone BiP for autophagy through p62 binding. Nature cell Biol.

[38] Cha-Molstad H, Yu JE, Feng Z, Lee SH, Kim JG, Yang P, Han B, Sung KW, Yoo YD, Hwang J, McGuire T, Shim SM, Song HD, Ganipisetti S, Wang N, Jang JM, Lee MJ, Kim SJ, Lee KH, Hong JT (2017). p62/SQSTM1/Sequestosome-1 is an N-recognin of the N-end rule pathway which modulates autophagosome biogenesis. Nat Commun.

[39] Yoo YD, Mun SR, Ji CH, Sung KW, Kang KY, Heo AJ, Lee SH, An JY, Hwang J, Xie XQ, Ciechanover A, Kim BY, Kwon YT (2018). N-terminal arginylation generates a bimodal degron that modulates autophagic proteolysis. Proc Natl Acad Sci USA.

[40] Ji CH, Kim HY, Heo AJ, Lee SH, Lee MJ, Kim SB, Srinivasrao G, Mun SR, Cha-Molstad H, Ciechanover A, Choi CY, Lee HG, Kim BY, Kwon YT (2019). The N-Degron Pathway Mediates ER-phagy. Mol Cell.

[41] Shim, S. M., Choi, H. R., Kwon, S. C., Kim, H. Y., Sung, K. W., Jung, E. J., Mun, S. R., Bae, T. H., Kim, D. H., Son, Y. S., Jung, C. H., Lee, J., Lee, M. J., Park, J. W., Kwon, Y. T. The Cys-N-degron pathway modulates pexophagy through the N-terminal oxidation and arginylation of ACAD10. Autophagy. 2022;1–20. 10.1080/15548627.2022.2126617.Advance online publication. 10.1080/15548627.2022.2126617 PMC10262816 36184612

[42] Lee YJ, Kim JK, Jung CH, Kim YJ, Jung EJ, Lee SH, Choi HR, Son YS, Shim SM, Jeon SM, Choe JH, Lee SH, Whang J, Sohn KC, Hur GM, Kim HT, Yeom J, Jo EK, Kwon YT (2022). Chemical modulation of SQSTM1/p62-mediated xenophagy that targets a broad range of pathogenic bacteria. Autophagy.

[43] Zhang Y, Mun SR, Linares JF, Ahn J, Towers CG, Ji CH, Fitzwalter BE, Holden MR, Mi W, Shi X, Moscat J, Thorburn A, Diaz-Meco MT, Kwon YT, Kutateladze TG (2018). ZZ-dependent regulation of p62/SQSTM1 in autophagy. Nat Commun.

[44] Ji CH, Kim HY, Lee MJ, Heo AJ, Park DY, Lim S, Shin S, Yang WS, Jung CA, Kim KY, Jeong EH, Park SH, Bin Kim S, Lee SJ, Na JE, Kang JI, Chi HM, Kim HT, Kim YK, Kim BY (2022). The AUTOTAC chemical biology platform for targeted protein degradation via the autophagy-lysosome system. Nat Commun.

[45] Ciechanover A, Kwon YT (2015). Degradation of misfolded proteins in neurodegenerative diseases: therapeutic targets and strategies. Exper Mol Med.

[46] Winslow AR, Chen CW, Corrochano S, Acevedo-Arozena A, Gordon DE, Peden AA, Lichtenberg M, Menzies FM, Ravikumar B, Imarisio S, Brown S, O'Kane CJ, Rubinsztein DC (2010). α-Synuclein impairs macroautophagy: implications for Parkinson's disease. J Cell Biol.

[47] Sarkar S, Olsen AL, Sygnecka K, Lohr KM, Feany MB (2021). α-synuclein impairs autophagosome maturation through abnormal actin stabilization. PLoS Genet.

[48] Bové J, Martínez-Vicente M, Vila M (2011). Fighting neurodegeneration with rapamycin: mechanistic insights. Nat Rev Neurosci.

[49] Decressac M, Mattsson B, Weikop P, Lundblad M, Jakobsson J, Björklund A (2013). TFEB-mediated autophagy rescues midbrain dopamine neurons from α-synuclein toxicity. Proc Natl Acad Sci USA.

[50] Deeg AA, Reiner AM, Schmidt F, Schueder F, Ryazanov S, Ruf VC, Giller K, Becker S, Leonov A, Griesinger C, Giese A, Zinth W (2015). Anle138b and related compounds are aggregation specific fluorescence markers and reveal high affinity binding to α-synuclein aggregates. Biochem Biophys Acta.

[51] Zhu M, Rajamani S, Kaylor J, Han S, Zhou F, Fink AL (2004). The flavonoid baicalein inhibits fibrillation of alpha-synuclein and disaggregates existing fibrils. J Biol Chem.

[52] Javed, H., Ojha, S. Therapeutic Potential of Baicalein in Parkinson’s Disease: Focus on Inhibition of α-Synuclein Oligomerization and Aggregation. In (Ed.), Synucleins - Biochemistry and Role in Diseases. IntechOpen. 2019. 10.5772/intechopen.83589

[53] Ahn JS, Lee JH, Kim JH, Paik SR (2007). Novel method for quantitative determination of amyloid fibrils of alpha-synuclein and amyloid beta/A4 protein by using resveratrol. Anal Biochem.

[54] Zhang LF, Yu XL, Ji M, Liu SY, Wu XL, Wang YJ, Liu RT (2018). Resveratrol alleviates motor and cognitive deficits and neuropathology in the A53T α-synuclein mouse model of Parkinson’s disease. Food Funct.

[55] Roy D, Kumar V, James J, Shihabudeen MS, Kulshrestha S, Goel V, Thirumurugan K (2015). Evidence that chemical chaperone 4-Phenylbutyric acid binds to human serum albumin at fatty acid binding sites. PLoS ONE.

[56] Inden M, Kitamura Y, Takeuchi H, Yanagida T, Takata K, Kobayashi Y, Taniguchi T, Yoshimoto K, Kaneko M, Okuma Y, Taira T, Ariga H, Shimohama S (2007). Neurodegeneration of mouse nigrostriatal dopaminergic system induced by repeated oral administration of rotenone is prevented by 4-phenylbutyrate, a chemical chaperone. J Neurochem.

[57] Burré J, Sharma M, Südhof TC (2014). α-Synuclein assembles into higher-order multimers upon membrane binding to promote SNARE complex formation. Proc Natl Acad Sci USA.

[58] Emin D, Zhang YP, Lobanova E, Miller A, Li X, Xia Z, Dakin H, Sideris DI, Lam JYL, Ranasinghe RT, Kouli A, Zhao Y, De S, Knowles TPJ, Vendruscolo M, Ruggeri FS, Aigbirhio FI, Williams-Gray CH, Klenerman D (2022). Small soluble α-synuclein aggregates are the toxic species in Parkinson’s disease. Nat Commun.

[59] Spillantini MG, Crowther RA, Jakes R, Hasegawa M, Goedert M (1998). alpha-Synuclein in filamentous inclusions of Lewy bodies from Parkinson's disease and dementia with lewy bodies. Proc Natl Acad Sci USA.

[60] Rideout HJ, Dietrich P, Wang Q, Dauer WT, Stefanis L (2004). alpha-synuclein is required for the fibrillar nature of ubiquitinated inclusions induced by proteasomal inhibition in primary neurons. J Biol Chem.

[61] Bae EJ, Lee HJ, Lee SJ (2016). Cell models to study cell-to-cell transmission of α-synuclein. Methods Mol Biol.

[62] Lee SJ, Desplats P, Sigurdson C, Tsigelny I, Masliah E (2010). Cell-to-cell transmission of non-prion protein aggregates. Nat Rev Neurol.

[63] Anderson JP, Walker DE, Goldstein JM, de Laat R, Banducci K, Caccavello RJ, Barbour R, Huang J, Kling K, Lee M, Diep L, Keim PS, Shen X, Chataway T, Schlossmacher MG, Seubert P, Schenk D, Sinha S, Gai WP, Chilcote TJ (2006). Phosphorylation of Ser-129 is the dominant pathological modification of alpha-synuclein in familial and sporadic Lewy body disease. J Biol Chem.

[64] Fujiwara H, Hasegawa M, Dohmae N, Kawashima A, Masliah E, Goldberg MS, Shen J, Takio K, Iwatsubo T (2002). alpha-Synuclein is phosphorylated in synucleinopathy lesions. Nat Cell Biol.

[65] Yoon YS, You JS, Kim TK, Ahn WJ, Kim MJ, Son KH, Ricarte D, Ortiz D, Lee SJ, Lee HJ (2022). Senescence and impaired DNA damage responses in alpha-synucleinopathy models. Exp Mol Med.

[66] Mashimo M, Onishi M, Uno A, Tanimichi A, Nobeyama A, Mori M, Yamada S, Negi S, Bu X, Kato J, Moss J, Sanada N, Kizu R, Fujii T (2021). The 89-kDa PARP1 cleavage fragment serves as a cytoplasmic PAR carrier to induce AIF-mediated apoptosis. J Biol Chem.

[67] Ganjam GK, Bolte K, Matschke LA, Neitemeier S, Dolga AM, Höllerhage M, Höglinger GU, Adamczyk A, Decher N, Oertel WH, Culmsee C (2019). Mitochondrial damage by α-synuclein causes cell death in human dopaminergic neurons. Cell Death Dis.

[68] Lee HJ, Kim C, Lee SJ (2010). Alpha-synuclein stimulation of astrocytes: Potential role for neuroinflammation and neuroprotection. Oxid Med Cell Longev.

[69] Lei Z, Cao G, Wei G (2019). A30P mutant α-synuclein impairs autophagic flux by inactivating JNK signaling to enhance ZKSCAN3 activity in midbrain dopaminergic neurons. Cell Death Dis.

[70] Choi I, Zhang Y, Seegobin SP, Pruvost M, Wang Q, Purtell K, Zhang B, Yue Z (2020). Microglia clear neuron-released α-synuclein via selective autophagy and prevent neurodegeneration. Nat Commun.

[71] Lee HJ, Suk JE, Patrick C, Bae EJ, Cho JH, Rho S, Hwang D, Masliah E, Lee SJ (2010). Direct transfer of alpha-synuclein from neuron to astroglia causes inflammatory responses in synucleinopathies. J Biol Chem.

[72] Sarkar S, Davies JE, Huang Z, Tunnacliffe A, Rubinsztein DC (2007). Trehalose, a novel mTOR-independent autophagy enhancer, accelerates the clearance of mutant huntingtin and alpha-synuclein. J Biol Chem.

[73] Sarkar S, Ravikumar B, Floto RA, Rubinsztein DC (2009). Rapamycin and mTOR-independent autophagy inducers ameliorate toxicity of polyglutamine-expanded huntingtin and related proteinopathies. Cell Death Differ.

[74] Gao J, Perera G, Bhadbhade M, Halliday GM, Dzamko N (2019). Autophagy activation promotes clearance of α-synuclein inclusions in fibril-seeded human neural cells. J Biol Chem.

[75] Malagelada C, Jin ZH, Jackson-Lewis V, Przedborski S, Greene LA (2010). Rapamycin protects against neuron death in in vitro and in vivo models of Parkinson’s disease. J Neurosci.

[76] Schaub T, Gürgen D, Maus D, Lange C, Tarabykin V, Dragun D, Hegner B (2019). mTORC1 and mTORC2 Differentially Regulate Cell Fate Programs to Coordinate Osteoblastic Differentiation in Mesenchymal Stromal Cells. Sci Rep.

[77] Kaldirim M, Lang A, Pfeiler S, Fiegenbaum P, Kelm M, Bönner F, Gerdes N (2022). Modulation of mTOR signaling in cardiovascular disease to target acute and chronic inflammation. Front Cardiovasc Med.

[78] Rusmini P, Cortese K, Crippa V, Cristofani R, Cicardi ME, Ferrari V, Vezzoli G, Tedesco B, Meroni M, Messi E, Piccolella M, Galbiati M, Garrè M, Morelli E, Vaccari T, Poletti A (2019). Trehalose induces autophagy via lysosomal-mediated TFEB activation in models of motoneuron degeneration. Autophagy.

[79] Redmann M, Wani WY, Volpicelli-Daley L, Darley-Usmar V, Zhang J (2017). Trehalose does not improve neuronal survival on exposure to alpha-synuclein pre-formed fibrils. Redox Biol.

[80] Turco E, Witt M, Abert C, Bock-Bierbaum T, Su MY, Trapannone R, Sztacho M, Danieli A, Shi X, Zaffagnini G, Gamper A, Schuschnig M, Fracchiolla D, Bernklau D, Romanov J, Hartl M, Hurley JH, Daumke O, Martens S (2019). FIP200 claw domain binding to p62 promotes autophagosome formation at ubiquitin condensates. Mol Cell.

[81] Turco E, Savova A, Gere F, Ferrari L, Romanov J, Schuschnig M, Martens S (2021). Reconstitution defines the roles of p62, NBR1 and TAX1BP1 in ubiquitin condensate formation and autophagy initiation. Nat Commun.

[82] Mazzulli JR, Zunke F, Isacson O, Studer L, Krainc D (2016). α-Synuclein-induced lysosomal dysfunction occurs through disruptions in protein trafficking in human midbrain synucleinopathy models. Proc Natl Acad Sci USA.

[83] Pan B, Li J, Parajuli N, Tian Z, Wu P, Lewno MT, Zou J, Wang W, Bedford L, Mayer RJ, Fang J, Liu J, Cui T, Su H, Wang X (2020). The Calcineurin-TFEB-p62 pathway mediates the activation of cardiac macroautophagy by proteasomal malfunction. Circ Res.

[84] Lindersson E, Beedholm R, Højrup P, Moos T, Gai W, Hendil KB, Jensen PH (2004). Proteasomal inhibition by alpha-synuclein filaments and oligomers. J Biol Chem.

[85] Dorsey ER, Sherer T, Okun MS, Bloem BR (2018). The Emerging Evidence of the Parkinson Pandemic. J Parkinsons Dis.

