# Correction: Targeted degradation of ⍺-synuclein aggregates in Parkinson’s disease using the AUTOTAC technology

**DOI:** 10.1186/s13024-023-00661-0

**Published:** 2023-09-29

**Authors:** Jihoon Lee, Ki Woon Sung, Eun-Jin Bae, Dabin Yoon, Dasarang Kim, Jin Saem Lee, Da-ha Park, Daniel Youngjae Park, Su Ran Mun, Soon Chul Kwon, Hye Yeon Kim, Joo-Ok Min, Seung-Jae Lee, Young Ho Suh, Yong Tae Kwon

**Affiliations:** 1 Cellular Degradation Biology Center, College of Medicine, Seoul National University, Seoul, 03080 Republic of Korea; 2 Department of Biomedical Sciences, College of Medicine, Seoul National University, Seoul, 03080 Republic of Korea; 3 AUTOTAC Bio Inc.,, Changkyunggung-Ro 254, Jongno-Gu, Seoul, 03077 Republic of Korea; 4 Neuroscience Research Institute, College of Medicine, Seoul National University, Seoul, 03080 Republic of Korea; 5 Department of Physical Education, Sejong University, Seoul, 05006 Republic of Korea; 6 Neuramedy Co. Ltd, Seoul, 04796 Republic of Korea; 7 Convergence Research Center for Dementia, Seoul National University Medical Research Center, Seoul, 03080 Republic of Korea; 8 Ischemic/Hypoxic Disease Institute, College of Medicine, Seoul National University, Seoul, 03080 Republic of Korea

Molecular Neurodegeneration (2023) 18:41

10.1186/s13024-023-00630-7.

The original article [1] contained errors in the Funding section. The correct Funding information can be viewed ahead:

**Funding**

This work was supported by the National Research Foundation of Korea (NRF) grants funded by the Ministry of Science, ICT and Future Planning (MSIP) (NRF-2020R1A5A1019023 to Y.T.K., NRF-2021R1A2B5B03002614 to Y.T.K., NRF-2020R1A5A1019023 to Y.H.S., and NRF-2022R1A2C1004913 to Y.H.S.), the Korea Dementia Research Project (HU21C0071 to Y.H.S.), and the Ministry of Education (RS-2023-00243474 to E.J.B.). This work was also supported by Korea Drug Development Fund (KDDF) (RS-2022-00166787 to K.W.S.).

