# Supplementary material for: Targeted degradation of ⍺-synuclein aggregates in Parkinson’s disease using the AUTOTAC technology
Source: Mol Neurodegener. 2023 Jun 24;18:41. doi: 10.1186/s13024-023-00630-7 (PMC10290391; doi:10.1186/s13024-023-00630-7)
Supplement: Supplementary file 7 — Additional file 7. Supplementary Methods. [file 13024_2023_630_MOESM7_ESM.docx]

**Supplementary Methods**

General experimental methods

^1^H NMR and ^13^C NMR spectra were recorded on Bruker Avance III 500 MHz, 400 MHz and Bruker Fourier 300 MHz and TMS was used as an internal standard.

LCMS was taken on a quadrupole Mass Spectrometer on Agilent 1260HPLC and 6120MSD (Column: C18 (50 × 4.6 mm, 5 μm) operating in ES (+) or (-) ionization mode; T = 30 ^o^C; flow rate = 1.5 mL/min; detected wavelength: 220/254 nm.

LC-HRMS was taken by 3 methods. One was taken on Agilent 6550 iFunnel Q-TOF (Column : ZORBAX RRHD SB-C18 (80Å, 2.1x100mm 1.8μm)) operating in ES (+) or (-) ionization mode ; T = 25 ^o^C ; flow rate = 0.5 mL/min ; detected wavelength : 254 nm.

*Synthesis of ATL7 (2-((4-(benzyloxy)-3-(3-phenylpropoxy)benzyl)amino)ethan-1-ol)*

Step 1: To a solution of 3,4-dihydroxybenzaldehyde (50 g, 0.36 mol) in ACN (0.7 L) were added NaHCO_3_ (39.5 g, 0.47mol) and BnBr (61.9 g, 0.36 mol). The mixture was stirred at 80 ^o^C for 16 hrs. The reaction was concentrated. The residue was quenched with HCl (1.5 L, 1N) and extracted with EA (1.2 L x3). The organic layer was washed with brine, dried over Na_2_SO_4_, filtered and concentrated. The residue was purified by silica gel, eluted with EA/PE (1:20~1:10) to afford 4-(benzyloxy)-3-hydroxybenzaldehyde (12 g, yield 14.6%) as a white solid. (TLC: EA/PE=1/5, Rf = 0.4).

LC-MS Calcd m/z for C_14_H_12_O_3_: 228.25; MS Found 229.1.

Step 2: To a solution of 4-(benzyloxy)-3-hydroxybenzaldehyde (12 g, 52.6 mmol) in THF (0.2 L) were added 3-phenylpropan-1-ol (8.59 g, 63.1 mmol), PPh_3_ (20.7 g, 78.9 mmol) and DIAD (16 g, 78.9 mmol). The mixture was **s**tirred at 65^o^C for 16 hrs. The reaction was concentrated and purification by silica gel, eluted with EA/PE (1:15~1:10) to afford 4-(benzyloxy)-3-(3-phenylpropoxy)benzaldehyde (8 g, yield 44%) as a yellow oil. (TLC: EA/PE=1/5, Rf = 0.6).

LC-MS Calcd m/z for C_23_H_22_O_3_: 346.43; MS Found 347.1.

Step 3: To a solution of 4-(benzyloxy)-3-(3-phenylpropoxy)benzaldehyde (40 g, 11.6 mmol) in MeOH (400 mL) was added 2-aminoethanol (10.6g g, 17.3 mmol). The mixture was **s**tirred at 65^o^C for 6 hrs. Then NaBH_4_ (8.8 g, 23mmol) was added to the reaction at 0^o^C. The resulting mixture was stirred at room temperature overnight. The reaction was concentrated, and purification by silica gel, eluted with DCM/MEOH (20:1) to afford **ATL7** (2-((4-(benzyloxy)-3-(3-phenylpropoxy)benzyl)amino)ethan-1-ol, 11g yield 24%) as a white solid. (TLC: DCM/MeOH=15/1, Rf = 0.5).

^1^H-NMR (DMSO-*d*_6_, 400 MHz): δ 7.47 (d, *J* = 7.2 Hz, 2H), 7.38 (d, *J* = 7.6 Hz, 2H), 7.26-7.33 (m, 3H), 7.17-7.22 (m, 3H), 6.97 (d, *J* = 8.4 Hz, 2H), 6.80 (d, *J* = 8.8 Hz, 1H), 5.09 (s, 2H), 4.47 (s, 1H), 3.97 (t, *J* = 6.0 Hz, 2H), 3.61 (s, 2H), 3.45 (t, *J* = 5.6 Hz, 2H), 2.76 (t, *J* = 7.6Hz, 2H), 2.54 (t, *J* = 6Hz, 2H), 1.98-2.05 (m, 2H).

LC-MS Calcd m/z for C_25_H_29_NO_3_: 391.21; MS Found: 392 [MS+1].

*Synthesis of ATL13 (2-((3-((4-fluorobenzyl)oxy)benzyl)amino)ethan-1-ol)*

Step 1: To a solution of 3-hydroxybenzaldehyde (20.0 g, 164 mmol, 1.0 eq) and 1-(bromomethyl)-4-fluorobenzene (40.3 g, 213 mmol, 1.3 eq) in DMF (100 mL) was add K_2_CO_3_ (33.9 g, 246 mmol, 1.5 eq) at 25 ^o^C. The mixture was stirred for 6 hrs at 25 ^o^C. The reaction was poured into water, then was filtered to give the 3-((4-fluorobenzyl)oxy)benzaldehyde (30.0 g, 79.6%). (TLC: PE/EA=5/1, R_f_=0.6)

^1^H-NMR (CDCl_3_, 400 MHz): δ 10.00 (s, 1H), 7.43-7.51 (m, 5H), 7.27 (s, 1H), 7.09-7.14 (m, 2H), 5.11 (s, 2H).

Step 2: To a solution of 3-((4-fluorobenzyl)oxy)benzaldehyde (30.0 g, 130 mmol, 1.00 eq) and 2-aminoethanol (7.96 g, 130 mmol, 1.00 eq) in MeOH (300 mL) was stirred for 1 hr at room temperature. The mixture was added NaBH(OAc)_3_ (83.0 g, 391 mmol, 3.00 eq) at room temperature, then stirred for 1 hr at room temperature. The reaction was quenched with saturated aqueous NH_4_Cl solution, washed by NaHCO_3_ solution, extracted with EA and concentrated. The crude was purified by column chromatography (DCM/MeOH=20/1~10/1) to give **ATL13 (**2-((3-((4-fluorobenzyl)oxy)benzyl)amino)ethan-1-ol, 11.0g, 30.7%) as off-white solid. (TLC: DCM/MeOH=10/1, R_f_=0.4)

^1^H-NMR (CDCl_3_, 400 MHz): δ 7.41-7.45 (m, 2H), 7.25-7.30 (m, 1H), 7.06-7.11 (m, 2H), 7.02 (s, 1H), 6.95-6.97 (m, 1H), 6.88-6.91 (m, 1H), 5.05 (s, 2H), 3.84 (s, 2H), 3.68-3.71 (m, 2H), 2.99 (s, 2H), 2.82-2.85 (m, 2H).

LCMS [mobile phase: from 95% water (0.05% FA) and 5% CH_3_CN/H_2_O (v/v=9/1, 0.05% FA) to 40% water (0.05% FA) and 60% CH_3_CN/H_2_O (v/v=9/1, 0.05% FA) in 6.0 min, finally under these conditions for 0.5 min.] purity is >99.1% (254 nm), Rt = 3.07 min; Mass Calcd.:275; MS Found: 276 [MS+1].

*Synthesis of ATL21* *((R)-1-(3,4-bis((4-fluorobenzyl)oxy)phenoxy)-3-((2hydroxyethyl) amino)propan-2-ol)*

**Step 1:** To a solution of 3,4-dihydroxybenzaldehyde (100 g, 724.6 mmol) in ACN (1000 mL) were added 1-(bromomethyl)-4-fluorobenzene (301.3 g, 1.59 mol) and K_2_CO_3_ (300 g, 2.17 mol). The mixture was stirred at 80 ^o^C for 16 hrs. Then the reaction was concentrated, the residue was purified by silica gel, eluted with EA/PE (1:15~1:8) to afford 3,4-bis((4-fluorobenzyl)oxy)benzaldehyde (187 g, 72.8 %) as a white solid.

^1^H-NMR (DMSO_d_6_, 400 MHz) δ (ppm) 9.83 (s, 1H), 7.55-7.48 (m, 6H), 7.30-7.20 (m, 5H), 5.25 (s, 2H), 5.19 (s, 2H).

**Step 2:** To a solution of 3,4-bis((4-fluorobenzyl)oxy)benzaldehyde (187 g, 526.7 mmol) in DCM (2000 mL) was added m-CPBA (126 g, 730.4 mmol). The mixture was stirred at room temperature for 16 hrs. Then the reaction was washed with saturated sodium bicarbonate solution, concentrated under vacuum. Then the crude product is added to methanol (1500 mL) and water (200 mL) was added KOH (58.9 g, 1.05 mol). The mixture was stirred at room temperature for 3 hrs. Then the reaction was filtered and the solid to dryness under vacuum. The crude compound was purified by silica gel, eluted with EA/PE (1:15~1:5) to afford 3,4-bis((4-fluorobenzyl)oxy)phenol (151 g, 83.7 %) as an off-white solid.

^1^H-NMR (DMSO_d_6_, 400 MHz) δ (ppm) 7.50-7.41 (m, 4H), 7.24-7.15 (m, 4H), 6.82 (d, J = 8.4 Hz, 1H), 6.49 (d, J = 2.4 Hz, 1H), 6.25 (dd, J = 8.8, 2.8 Hz, 1H), 5.04 (s, 2H), 4.95 (s, 2H)

**Step 3:** To a solution of 3,4-bis((4-fluorobenzyl)oxy)phenol (45.8 g, 134 mmol) in EtOH (500 mL) were added water (25 mL) and KOH (17.2 g, 307 mmol). Then (*R*)-2-(chloromethyl)oxirane (37 g, 400 mmol) was added to the reaction. The resulting mixture was stirred at room temperature for 16 hrs. Then the reaction was quenched by addition water, extracted with EA. The organic layer was washed with brine, dried over Na_2_SO_4_, filtered and concentrated. The residue was purified by silica gel, eluted with EA/PE (1:15~1:10) to afford (*R*)-2-((3,4-bis((4-fluorobenzyl)oxy)phenoxy)methyl)oxirane (26 g, 48.7 %) as a white solid.

^1^H-NMR (DMSO_d_6_, 400 MHz) δ (ppm) 7.51-7.43 (m, 4H), 7.25-7.16 (m, 4H), 6.94 (d, J = 9.2 Hz, 1H), 6.73 (d, J = 2.8 Hz, 1H), 6.45 (dd, J = 8.8, 2.8 Hz, 1H), 5.10 (s, 2H), 5.00 (s, 2H), 4.24 (dd, J = 11.2, 2.8 Hz, 1H), 3.75 (dd, J = 11.2, 6.4 Hz, 1H), 3.30-3.28 (m, 1H), 2.83 (t, J = 5.2 Hz, 1H), 2.68 (dd, J = 5.2, 2.8 Hz, 1H)

**Step 4:** To a solution of (*R*)-2-((3,4-bis((4-fluorobenzyl)oxy)phenoxy)methyl)oxirane (10.0 g, 25.1 mmol) and 2-aminoethanol (3.07 g, 50.3 mmol) in MeOH (100 mL) was stirred overnight at 50 ^o^C. The mixture was concentrated. Another 20 g batch was carried out as the above procedure. The crude was purified by prep-HPLC, then concentrated to remove acetonitrile, and added the NaHCO_3_ saturated solution to adjust pH to 7-8. The solution was filtered and washed by water 3 times to give **ATL21** ((*R*)-1-(3,4-bis((4-fluorobenzyl)oxy)phenoxy)-3-((2-hydroxyethyl)amino)propan-2-ol , 10 g, 86.7 %) as a white solid.

^1^H-NMR (CDCl_3_, 400 MHz) δ (ppm) 7.40-7.33 (m, 4H), 7.06-6.99 (m, 4H), 6.83 (d, *J* = 8.8 Hz, 1H), 6.57 (d, *J* = 2.8 Hz, 1H), 6.38 (dd, J = 9.2, 3.2 Hz, 1H), 5.04 (s, 2H), 4.99 (s, 2H), 4.07 (brs, 1H), 3.90 (d, *J* = 4.4 Hz, 2H), 3.70 (s, 2H), 2.88-2.77 (m, 7H) ; LC-MS Calcd m/z for C_25_H_27_F_2_NO_5_ [M+H]^+^ 459.18 Found 460.90.

*Synthesis of ATC161* *(3-(3-(benzo[d][1,3]dioxol-5-yl)-1H-pyrazol-5-yl)-N-(2-(2-(2-((3-((4-fluorobenzyl)oxy)benzyl)amino)ethoxy)ethoxy)ethyl)aniline*

Step 1: To a solution of 1-(benzo[*d*][1,3]dioxol-5-yl)ethan-1-one (20.0 g, 122 mmol) and NaH (6.10 g, 152 mmol, 60% in mineral oil) in dry dimethylsulfoxide (120 mL) was stirred at 15 ^o^C for 30 min. Then a solution of methyl 3-bromobenzoate (32.8 g, 152 mmol) in dimethylsulfoxide (60 mL) was added at 20 ^o^C. The resulting mixture was stirred at 25 ^o^C for 2 hours. The reaction was quenched with saturated aq. NH_4_Cl solution. The mixture was poured into water and petroleum ether and stirred for 30 min at r.t. Then filtered to give 1-(benzo[*d*][1,3]dioxol-5-yl)-3-(3-bromophenyl)propane-1,3-dione (35.0 g, crude) as yellow solid.

^1^H-NMR (CDCl_3_, 400 MHz) δ (ppm) 8.07 (s, 1H), 7.88 (d, J = 7.6 Hz, 1H), 7.69-7.59 (m, 2H), 7.46 (s, 1H), 7.37-7.30 (m, 1H), 6.90 (d, J = 8 Hz, 1H), 6.69 (s, 1H), 6.07 (s, 2H).

LC-MS Calcd m/z for C_16_H_11_BrO_4_ [M+H]^+^ 347.00, Found 346.98.

Step 2: To a solution of 1-(benzo[*d*][1,3]dioxol-5-yl)-3-(3-bromophenyl)propane-1,3-dione (35.0 g, 101 mmol) and 98% N_2_H_4_.H_2_O (5.80 g, 116 mmol) in ethanol (600 mL) was refluxed for 2 hours. The mixture was cooled to r.t and filtered to give 3-(benzo[*d*][1,3]dioxol-5-yl)-5-(3-bromophenyl)-1*H*-pyrazole (25.0 g, 63 %) as off-white solid.

^1^H-NMR (DMSO_d_6_, 400 MHz) δ (ppm) 13.08 (s, 1H), 8.02 (s, 1H), 7.82 (d, J = 7.6 Hz, 1H), 7.52-7.32 (m, 4H), 7.08 (s, 1H), 6.98 (d, J = 8 Hz, 1H), 6.05 (brs, 2H); LC-MS Calcd m/z for C_16_H_11_BrN_2_O_2_ [M+H]^+^ 344.00 Found 343.00

Step 3: To a solution of 3-(benzo[*d*][1,3]dioxol-5-yl)-5-(3-bromophenyl)-1*H*-pyrazole (25.0 g, 72.9 mmol) and 2,2'-(ethane-1,2-diylbis(oxy))diethanamine (32.4 g, 219 mmol) in 1,4-dioxane (250 mL) was added NaH (8.75 g, 219 mmol, 60% in mineral oil) slowly at 25 ^o^C. Then added Pd_2_(dba)_3_ (2.5 g, 2.73 mmol) and 2,2′-bis(diphenylphosphino)-1,1′-binaphthyl (BINAP, 5 g, 8.05 mmol) at 25 ^o^C under N_2_. The mixture was stirred overnight at 100 ^o^C under N_2_. The reaction was cooled to r.t, then quenched with water, extracted with ethyl acetate and concentrated. The crude was purified by column chromatography (dichloromethane/methanol=50/1~5/1) to give the *N*-(2-(2-(2-aminoethoxy)ethoxy)ethyl)-3-(3-(benzo[*d*][1,3]dioxol-5-yl)-1*H*-pyrazol-5-yl)aniline (12.0 g, 40 %).

^1^H-NMR (CDCl_3_, 400 MHz) δ (ppm) 7.29 (s, 2H), 7.22-7.20 (m, 1H), 7.01-6.99 (m, 2H), 6.83 (d, J = 8 Hz, 1H), 6.69 (s, 1H), 6.58 (d, J = 8 Hz, 1H), 5.97 (s, 1H), 3.73-3.71 (m, 2H), 3.66-3.55 (m, 6H), 3.47 (s, 2H), 3.29-3.27 (m, 2H), 3.01-2.98 (m, 2H); LC-MS Calcd m/z for C_22_H_26_N_4_O_4_ [M+H]^+^ 411.20, Found 410.20

Step 4: To a solution of *N*-(2-(2-(2-aminoethoxy)ethoxy)ethyl)-3-(3-(benzo[*d*][1,3]dioxol-5-yl)-1*H*-pyrazol-5-yl)aniline (12.0 g, 29.3 mmol) and 3-((4-fluorobenzyl)oxy)benzaldehyde (**1-1**, 6.73 g, 29.3 mmol) in methanol (120 mL) was stirred for 1 hour at r.t. The mixture was added NaBH(OAc)_3_ (18.6 g, 87.8 mmol) at r.t, then stirred for 1 hour at r.t. The reaction was quenched with saturated aq. NH_4_Cl solution, extracted with ethyl acetate and concentrated. Another 3 g batch was carried out as the above procedure. The crude was purified by prep-HPLC to give **ATC161** (3-(3-(benzo[*d*][1,3]dioxol-5-yl)-1*H*-pyrazol-5-yl)-*N*-(2-(2-(2-((3-((4-fluorobenzyl)oxy)benzyl)amino)ethoxy)ethoxy)ethyl)aniline, 10.0g, 44 %) as yellow oil.

^1^H-NMR (DMSO_d_6_, 500 MHz) δ (ppm) 13.14 (s, 1H), 7.51 – 7.44 (m, 2H), 7.39 (d, J = 1.7 Hz, 1H), 7.34 (d, J = 7.8 Hz, 1H), 7.26 – 7.16 (m, 3H), 7.11 (t, J = 7.8 Hz, 1H), 7.05 – 7.00 (m, 2H), 7.00 – 6.94 (m, 3H), 6.91 (d, J = 7.6 Hz, 1H), 6.88 – 6.83 (m, 1H), 6.57 (d, J = 7.5 Hz, 1H), 6.05 (s, 2H), 5.64 (brs, 1H), 5.04 (s, 2H), 3.72 (s, 2H), 3.61 – 3.48 (m, 9H), 3.24 (q, J = 5.6 Hz, 2H), 2.67 (t, J = 5.7 Hz, 2H) ; ^13^C-NMR (DMSO_d_6,_ 125 MHz) δ (ppm) 162.71, 160.77, 158.26, 149.04, 147.72, 133.38, 133.36, 129.95, 129.89, 129.24, 120.68, 118.75, 115.31, 115.14, 114.55, 113.13, 108.58, 105.55, 101.09, 99.07, 69.69, 69.68, 69.52, 69.06, 68.35, 54.95, 52.41, 47.68, 42.67 ; HRMS Calcd m/z for C_36_H_37_FN_4_O_5_ [M+H]^+^ 625.2820 Found 625.2821.
